# Investigation of the *AQP* Family in Soybean and the Promoter Activity of *TIP2;6* in Heat Stress and Hormone Responses

**DOI:** 10.3390/ijms20020262

**Published:** 2019-01-10

**Authors:** Zhi-Juan Feng, Na Liu, Gu-Wen Zhang, Fu-Ge Niu, Sheng-Chun Xu, Ya-Ming Gong

**Affiliations:** 1 Institute of Vegetables, Zhejiang Academy of Agricultural Sciences, Hangzhou 310021, China; zhijuanke@163.com (Z.-J.F.); ln200811@163.com (N.L.); zhangguwen@126.com (G.-W.Z.); scxu@mail.zaas.ac.cn (S.-C.X.); 2 Food Safety Key Lab of Zhejiang Province, The School of Food Science and Biotechnology, Zhejiang Gongshang University, Hangzhou 310018, China; niufg123@hotmail.com

**Keywords:** soybean, aquaporin, heat stress, hormone cues, transcript expression, promoter, activated GUS, *GmTIP2;6*

## Abstract

Aquaporins (AQPs) are one diverse family of membrane channel proteins that play crucial regulatory roles in plant stress physiology. However, the heat stress responsiveness of *AQP* genes in soybean remains poorly understood. In this study, 75 non-redundant AQP encoding genes were identified in soybean. Multiple sequence alignments showed that all GmAQP proteins possessed the conserved regions, which contained 6 trans-membrane domains (TM1 to TM6). Different GmAQP members consisted of distinct Asn-Pro-Ala (NPA) motifs, aromatic/arginine (ar/R) selectivity filters and Froger’s positions (FPs). Phylogenetic analyses distinguished five sub-families within these GmAQPs: 24 GmPIPs, 24 GmTIPs, 17 GmNIPs, 8 GmSIPs, and 2 GmXIPs. Promoter *cis*-acting elements analyses revealed that distinct number and composition of heat stress and hormone responsive elements existed in different promoter regions of *GmAQPs*. QRT-PCR assays demonstrated that 12 candidate *GmAQPs* with relatively extensive expression in various tissues or high expression levels in root or leaf exhibited different expression changes under heat stress and hormone cues (abscisic acid (ABA), l-aminocyclopropane-l-carboxylic acid (ACC), salicylic acid (SA) and methyl jasmonate (MeJA)). Furthermore, the promoter activity of one previously functionally unknown *AQP* gene-*GmTIP2;6* was investigated in transgenic *Arabidopsis* plants. The beta-glucuronidase (GUS) activity driven by the promoter of *GmTIP2;6* was strongly induced in the heat- and ACC-treated transgenic plants and tended to be accumulated in the hypocotyls, vascular bundles, and leaf trichomes. These results will contribute to uncovering the potential functions and molecular mechanisms of soybean *GmAQPs* in mediating heat stress and hormone signal responses.

## 1. Introduction

Aquaporins (AQPs), known as membrane channel proteins, transport water as well as other small solutes (AQPs). AQPs consist of six trans-membrane (TM) helical domains with two cytoplasmic termini. AQPs contain two putative Asn-Pro-Ala (NPA) motifs located in the TM helices, aromatic/arginine (ar/R) regions and Froger’s positions (FPs) [1]. AQPs belong to an ancient, abundant, and highly diversified protein super-family [2,3,4,5,6,7]. Based on the protein sequence homology and membrane localization, plant AQPs are divided into five sub-families: plasma membrane intrinsic proteins (PIPs), tonoplast intrinsic proteins (TIPs), NOD26-like intrinsic proteins (NIPs), small basic intrinsic proteins (SIPs), and the unrecognized X intrinsic proteins (XIPs) [8].

*AQPs* extensively participated in plant physiological processes under variable environmental stresses [9,10]. Transcript profiles or gene function analyses of *AQPs* from many plant species, such as *Arabidopsis*, rice, barley, sorghum, cassava, soybean, and potato, demonstrated that they were associated with drought, cold, salt, silicon, or ABA stress [11,12,13,14,15,16,17]. Recently, several publications reported that *AQPs* were involved in response to heat stress. In wheat, *TaTIPs* respond to the combined heat and drought stresses, based on the representation of expressed sequence tags (ESTs) in wheat grain-related cDNA libraries [18]. In rhododendrons, the transcripts of *Rc/RpPIP2s* were associated with thermonasty (leaf-curling) under freezing-rewarming cycles [19]. In strawberries, heat stress induced the gene expression of *FaPIPs* [20]. In *Setaria viridis*, heat stress activated the expression of *SvPIPs* [21]. In *Rhazya stricta*, heat stress enhanced the abundant transcripts of *RsPIPs* and *RsTIPs* [22]. In *Arabidopsis*, *AtPIPs* were highly up-regulated due to combined heat-drought stress [23]. Nevertheless, functions and mechanisms of soybean *AQPs* in heat stress tolerance remain obscure. 

Soybean is an important economic crop and a staple food for people worldwide. Extreme heat conditions significantly reduce the productivity and weaken the global food security of soybean, especially given the growing impacts of climate changes [24,25,26,27]. In previous reports [28,29], 66 and 72 *GmAQP* members were identified from soybean, respectively, and the expression patterns of *GmAQPs* under drought or silicon stress were analyzed. However, whether *GmAQP* genes respond to heat stress in soybean remains poorly understood. This study focused on the investigation of correlation among expression of *GmAQPs*, heat stress, and different hormone signals. Gene numbers of *GmAQPs* were finally determined based on the recently-updated genome database Phytozome V12.1. Protein feature, sequence phylogeny, chromosomal location, and promoter elements of *GmAQPs* were also analyzed. Expressional patterns of 12 candidate *GmAQPs* with relatively extensive expression in various tissues or high expression levels in root or leaf in response to heat stress and different hormone treatments (abscisic acid (ABA), l-aminocyclopropane-l-carboxylic acid (ACC), salicylic acid (SA) and methyl jasmonate (MeJA)) were examined using quantitative real-time PCR (qRT-PCR). Additionally, the promoter activity of *GmTIP2;6* was assessed using the reporter *beta-glucuronidase* (*GUS*) gene in transgenic *Arabidopsis* plants under both control and stressful conditions. These results will provide foundation for further elucidating the molecular mechanism of soybean *GmAQPs* in modulating plant thermo-tolerance.

## 2. Results

### 2.1. Identification of the Soybean AQP Family

Based on HMM, KEGG, and protein BLAST searches, a total of 75 *GmAQP* members were identified and annotated from the recently-updated soybean genome database Phytozome V12.1 (Table 1; Dataset S1). Among them, the length of the *GmAQP* CDS sequence ranged from 693 bp of *GmSIP2;1* to 1092 bp of *GmPIP1;9*. The identified *GmAQP* genes encoded proteins ranging from 230 amino acids of *GmSIP2;1* to 363 amino acids of *GmPIP1;9*. Similarly, the molecular masses of the GmAQP proteins varied from 24.08 KDa of GmTIP2;7 to 39.41 KDa of GmPIP1;9 and the *pI* ranged from 5.08 of GmTIP2;1 and GmTIP2;2 to 10.01 of GmTIP1;9. Compared with previous reports [28,29], we newly identified 17 *GmAQPs* (*GmAQP9*, *GmAQP10*, *GmAQP13*, *GmAQP14*, *GmAQP18*, *GmAQP19*, *GmAQP20*, *GmAQP21*, *GmAQP22*, *GmAQP23*, *GmAQP24*, *GmAQP34*, *GmAQP54*, *GmAQP56*, *GmAQP57*, *GmAQP58* and *GmAQP74*) and 3 *GmAQPs* (*GmAQP9*, *GmAQP10* and *GmAQP34*) not previously observed, respectively.

In phosphorylation site analyses, 72% of GmAQPs were found to contain all three phosphorylation sites (Ser, Thr and Tyr). Among them, Ser and Thr phosphorylation sites were found in 7 GmTIPs (GmTIP1;1, GmTIP1;2, GmTIP1;3, GmTIP1;6, GmTIP1;9, GmTIP1;10 and GmTIP4;1), 4 GmNIPs (GmNIP5;2, GmNIP6;1, GmNIP6;2 and GmNIP6;3), and 2 GmSIPs (GmSIP2;1 and GmSIP2;2). Ser and Tyr phosphorylation sites were present in GmPIP2;1, GmPIP2;2, and GmTIP3;2. Thr and Tyr phosphorylation sites were distributed in GmTIP1;7 and GmTIP1;8. Ser and Tyr phosphorylation sites existed in GmTIP3;3. Thr phosphorylation sites were located in GmSIP1;1 and GmSIP1;2.

### 2.2. Key Structural Features of the AQP Proteins

To understand the possible physiological role and substrate specificity of soybean AQP proteins, the TM domains, NPA motifs, ar/R selectivity filters, and FPs were investigated (Table 2; Figures S1–S6). Protein structure analyses supported that all GmAQP proteins possessed the typically conserved regions, which contained 6 TM domains (TM1 to TM6) (Figure S1). All GmPIPs and GmTIPs contained two conserved NPA motifs in LB and LE. In GmNIPs, the first NPA showed the same sequence as in PIPs and TIPs, except for GmNIP5;2, where A was replaced by S. The second NPA motif showed an A to V substitution in four NIPs (GmNIP1;6, GmNIP5;2, GmNIP6;2, and GmNIP6;3). GmSIPs showed a second NPA motif completely conserved with the other members. Instead, all of the first NPA motifs showed the replacement of A by T (GmSIP1;1, GmSIP1;2, GmSIP1;3, and GmSIP1;4), A by S (GmSIP1;5 and GmSIP1;6), and A by L (GmSIP2;1 and GmSIP1;2). In GmXIPs, A in the first NPA of GmXIP1;1 was changed to I, and N in the second NPA was changed to S. The second NPA of GmXIP1;2 was completely conserved, while the N and A residues in the first NPA were replaced by S and V.

The ar/R positions (H2, H5, LE1, and LE2) of GmAQPs showed increased sub-family specificity compared to the two NPA motifs. In GmPIPs, all selectivity filters were F-H-T-R. In GmTIPs, the ar/R positions were formed by H/S in H2, I/V in H5, A/G in LE1, and V/A/R/L/C in LE2. In GmTIPs, these selectivity filters were constituted by W/A/T/N/S/G in H2, V/S/I in H5, A/G/S in LE1, and R in LE2. GmSIPs showed I/V/N/S in H2, I/V/N/H in H5, P/G in LE1, and F/A/S in LE2. The ar/R sites in GmXIPs were quite homogeneous, with V in H2 and H5, A/V in LE1, and R in LE2. The FPs (P1, P2, P3, P4 and P5) of GmAQPs exhibited divergent combinations, such as E/Q/M-S-A-F/Y-W for GmPIPs, T/S/V-S/A/T/C-A/S-F/Y-W for GmTIPs, F/Y/L-T/S-A-Y-W/V/I/L/M/F for GmNIPs, F/N/S-M/L-A-Y-W for GmSIPs, and E/D-C-A-F-W for GmXIPs.

### 2.3. Chromosome Distribution of the AQP Genes

**Figure 1:**
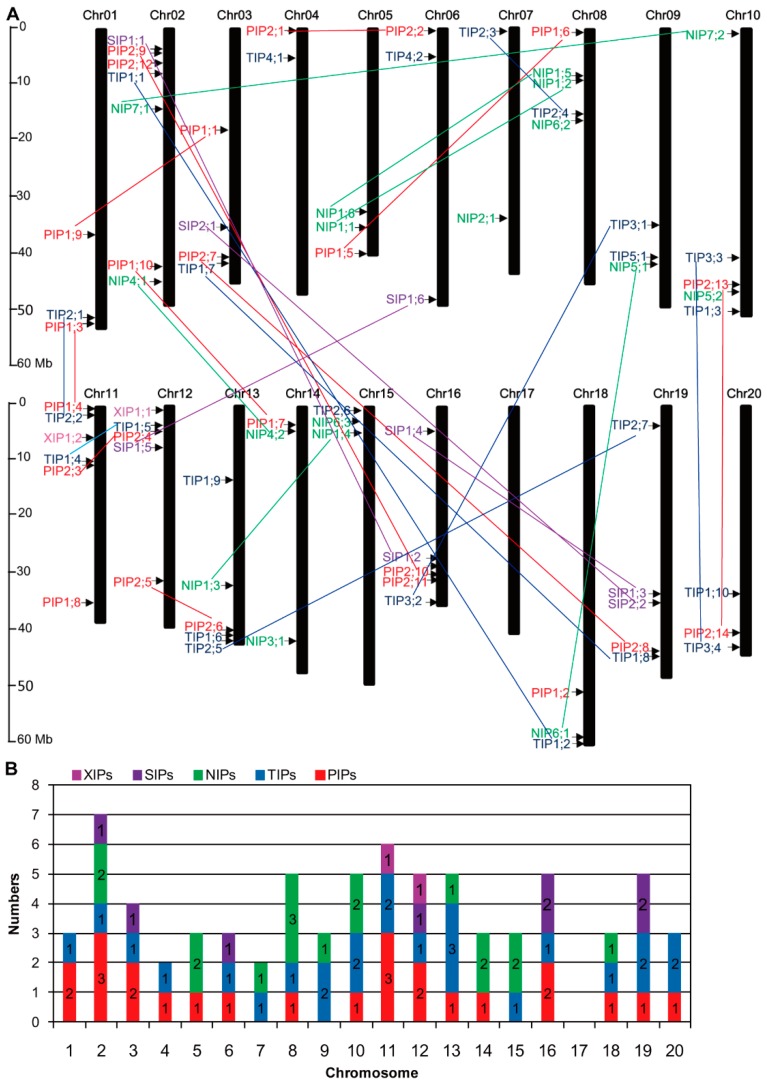
Chromosomal distribution of soybean *AQP* genes. (**A**) Graphical representation of physical locations for each *AQP* gene on soybean chromosomes (numbered Chr01–20). The scale on the left indicated the genomic length in megabases (Mb). *PIPs*, *TIPs*, *NIPs*, *XIPs* and *SIPs* were indicated with red, blue, green, purple and pink fonts, respectively. Lines represented putative gene duplications. (**B**) Numbers of *PIPs*, *TIPs*, *NIPs*, *XIPs* and *SIPs* on each soybean chromosome.

The genomic distribution of each soybean *AQP* was investigated, as indicated in Figure 1. Seventy-five *GmAQPs* were mapped on 19 chromosomes (Figure 1A). Among them, *GmAQPs* on chromosomes 2, 4, 6, 8, 11, 12, 14, and 15 exhibited the tendency to scatter closely to the upper end of the arm, while *GmAQPs* on chromosomes 1, 3, 5, 9, 10, 13, 16, 18, 19, and 20 tended to scatter closely to the lower end of the arm. Gene numbers on chromosomes 2 (7 loci each chromosome) were the maximum, whereas the gene numbers on chromosomes 4 and 7 (2 loci each chromosome) were the minimum (Figure 1B). *PIPs* and *TIPs* scattered extensively over the soybean chromosomes. While *NIPs* seemed to locate on chromosomes 2, 5, 7, 8, 9, 10, 13, 14, 15, and 18, *SIPs* were positioned on chromosomes 2, 3, 6, 12, 16 and 19. *XIPs* were resided on chromosomes 11 and 12 (Figure 1B).

To further understand the expansion mechanism of *GmAQPs*, the gene duplication events were analyzed. Two duplication events (*GmPIP2;9/PIP2;12* and *GmPIP2;10/PIP2;11*) within the same chromosome and twenty-eight duplication events (*GmPIP1;1/PIP1;9*, *GmPIP1;3/PIP1;4*, *GmPIP1;5/PIP1;6*, *GmPIP1;7/PIP1;10*, *GmPIP2;1/PIP2;2*, *GmPIP2;3/PIP2;4*, *GmPIP2;5/PIP2;6*, *GmPIP2;7/PIP2;8*, *GmPIP2;9/PIP2;10*, *GmPIP2;13/PIP2;14*, *GmTIP1;1/TIP1;2*, *GmTIP1;4/TIP1;5*, *GmTIP1;7/TIP1;8*, *GmTIP2;1/TIP2;2*, *GmTIP2;3/TIP2;4*, *GmTIP2;5/TIP2;7*, *GmTIP3;1/TIP3;2*, *GmTIP3;3/TIP3;4*, *GmNIP1;1/NIP1;2*, *GmNIP1;3/NIP1;4*, *GmNIP1;5/NIP1;6*, *GmNIP4;1/NIP4;2*, *GmNIP5;1/NIP6;1*, *GmNIP7;1/NIP7;2*, *GmSIP1;1/SIP1;2*, *GmSIP1;3/SIP1;4*, *GmSIP1;5/SIP1;6* and *GmSIP2;1/SIP2;2*) between different chromosomes were identified, respectively.

### 2.4. Evolutionary Characterization of the AQP Genes

**Figure 2:**
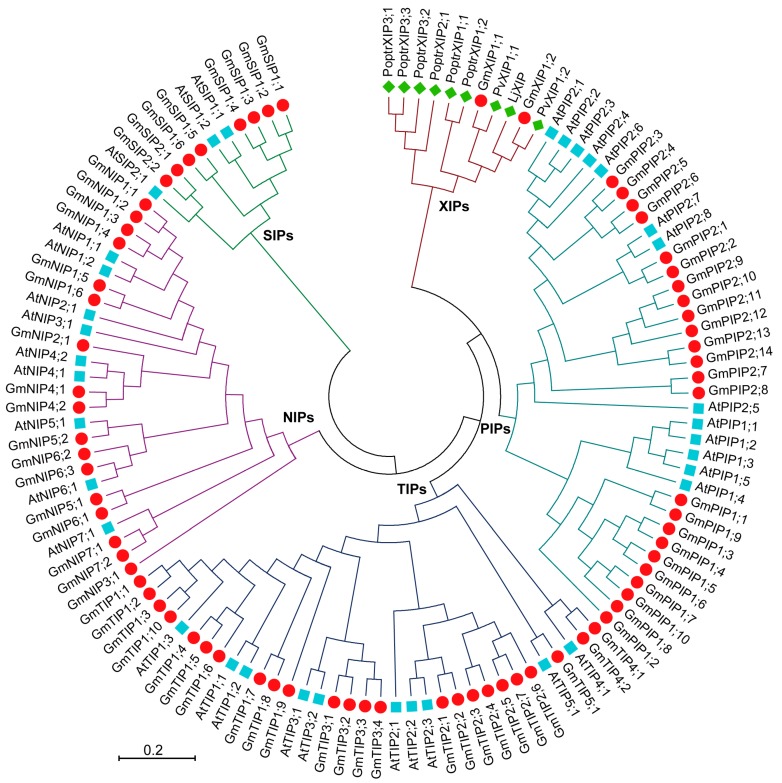
Phylogenetic relationships of AQP proteins from soybean, *Arabidopsis*, *Phaseolus vulgaris*, *Populus trichocarpa* and *Lotus japonicus*. The five sub-families were indicated with different colors. The red spots represent soybean AQPs. The blue squares represent *Arabidopsis* AQPs. The green diamonds represent *Phaseolus vulgaris*, *Populus trichocarpa* and *Lotus japonicus* XIPs.

To investigate the classification and evolutionary relationship of soybean AQP proteins, the phylogenetic tree was constructed with the full-length GmAQP protein sequences from *Arabidopsis*, *Phaseolus vulgaris*, *Populus trichocarpa*, and *Lotus japonicus* (Dataset S1; Dataset S2). Soybean *AQPs* grouped into five sub-families (*PIPs*, 24; *TIPs*, 24; *NIPs*, 17; *SIPs*, 8; *XIPs*, 2) (Figure 2). Among the *PIP* sub-family, 24 members were divided into two groups: *PIP1* with 10 members and *PIP2* with 14 members. Five groups were found for the *TIP* sub-family (*TIP1* to *TIP5*), with 10 members in the *TIP1* group, 7 members in the *TIP2* group, 4 members in the *TIP3* group, 2 members in the *TIP4* group, and 1 member in the *TIP5* group. Seven groups belonged to the *NIP* sub-family (*NIP1* to *NIP7*), with 6 members in the *NIP1* group, 1 member in the *NIP2* group, 1 member in the *NIP3* group, 2 members in the *NIP4* group, 2 members in the *NIP5* group, 3 members in the *NIP6* group, and 2 members in the *NIP7* group. The *SIP* sub-family were composed of two groups (*SIP1* and *SIP2*), with 6 members in the *SIP1* group and 2 members in the *SIP2* group. Only one group was identified for the *XIP* sub-family (*XIP1*), with 2 members.

Moreover, the putative orthologues of soybean *GmAQPs* with known *Arabidopsis AtAQPs* were identified. For *PIP* sub-family genes, *GmPIP2;1* and *GmPIP2;2* were the best orthology matches of *Arabidopsis AtPIP2;7* and *AtPIP2;8*; *GmPIP2;3*, *GmPIP2;4*, *GmPIP2;5,* and *GmPIP2;6* were the most homogeneous genes of *Arabidopsis AtPIP2;1*, *AtPIP2;2*, *AtPIP2;3*, *AtPIP2;4*, and *AtPIP2;6*. For *TIP* sub-family genes, *GmTIP1;1*, *GmTIP1;2*, *GmTIP1;3*, and *GmTIP1;10* exhibited the closest relationship with *Arabidopsis AtTIP1;3*. *GmTIP2;1* and *GmTIP2;2* clustered closely with *Arabidopsis AtTIP2;2* and *AtTIP2;3*. *GmTIP3;1*, *GmTIP3;2*, *GmTIP3;3*, and *GmTIP3;4* shared a fairly close evolutionary relationship with *Arabidopsis AtTIP3;1* and *AtTIP3;2*. *GmTIP4;1* and *GmTIP4;2* were phylogenetically closest to *Arabidopsis AtTIP4;1*. *GmTIP5;1* was in the same evolutionary clade with *Arabidopsis AtTIP5;1*. For *NIP* sub-family genes, *GmNIP1;1*, *GmNIP1;2*, *GmNIP1;3*, and *GmNIP1;4* showed the closest relationship with *Arabidopsis AtNIP1;1* and *AtNIP1;2*. *GmNIP4;1* and *GmNIP4;2* gathered closely with *Arabidopsis AtNIP4;1* and *AtNIP4;2*. *GmNIP5;2* was highly homologous with *Arabidopsis AtNIP5;1*. *GmNIP7;1* and *GmNIP7;2* grouped closely with *Arabidopsis AtNIP7;1*. For *SIP* sub-family genes, *GmSIP1;1*, *GmSIP1;2*, *GmSIP1;3*, and *GmSIP1;4* were the potential orthologs with *Arabidopsis AtSIP1;1* and *AtSIP1;2*. *GmSIP2;1* and *GmSIP2;2* were highly homologous with *Arabidopsis AtSIP2;1*.

### 2.5. Expression Profiles of the AQP Genes in Different Tissues

**Figure 3:**
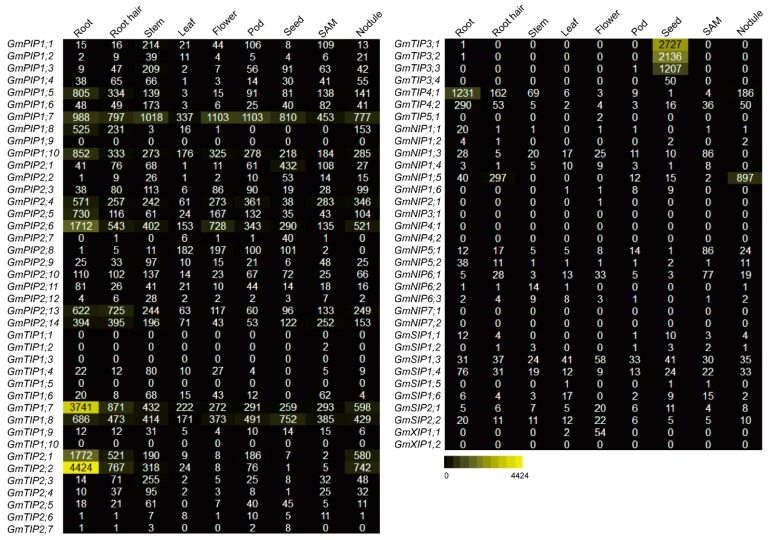
Expression profiles of soybean *AQP* genes in different tissues. Different expression levels in nine tissues (leaf, stem, root, flower, seed, root hair, pod, shoot apical meristem (SAM), and nodule.) were indicated in color using the RNA-seq data.

To examine the tissue expression profiles of the soybean *AQP* genes, the RNA-seq data were retrieved from available soybean database Phytozome V12.1. For different *GmAQP* members, different expression profiles were represented by different colors (Figure 3). Among them, one *PIP* gene *GmPIP1;9*, four *TIP* genes (*GmTIP1;1*, *GmTIP1;3*, *GmTIP1;5*, and *GmTIP1;10*), five *NIP* genes (*GmNIP3;1*, *GmNIP4;1*, *GmNIP4;2*, *GmNIP7;1*, and *GmNIP7;2*), and one *XIP* gene *GmXIP1;2* did not express in any tested tissues of soybean. In contrast, *GmPIP1;7*, *GmPIP1;10*, *GmPIP2;4*, *GmPIP2;6*, *GmTIP1;7*, and *GmTIP1;8* were highly expressed in all the investigated tissues. Additionally, for different tissues, the expression level analyses indicated significant differentiation among different *GmAQP* members. *GmPIP1;5*, *GmPIP1;8*, *GmPIP2;13*, *GmPIP2;14*, *GmTIP2;1*, *GmTIP2;2*, and *GmTIP4;1* were significantly expressed in roots. *GmPIP2;8* was highly expressed in leaves and flowers. *GmPIP2;1*, *GmTIP3;1*, *GmTIP3;2*, and *GmTIP3;3* were mainly expressed in seeds. *GmTIP2;3* was highly expressed in stems. *GmNIP1;5* was significantly expressed in root hairs and nodules.

### 2.6. Expression Profiles of the Candidate AQP Genes in Response to Heat Stress

**Figure 4:**
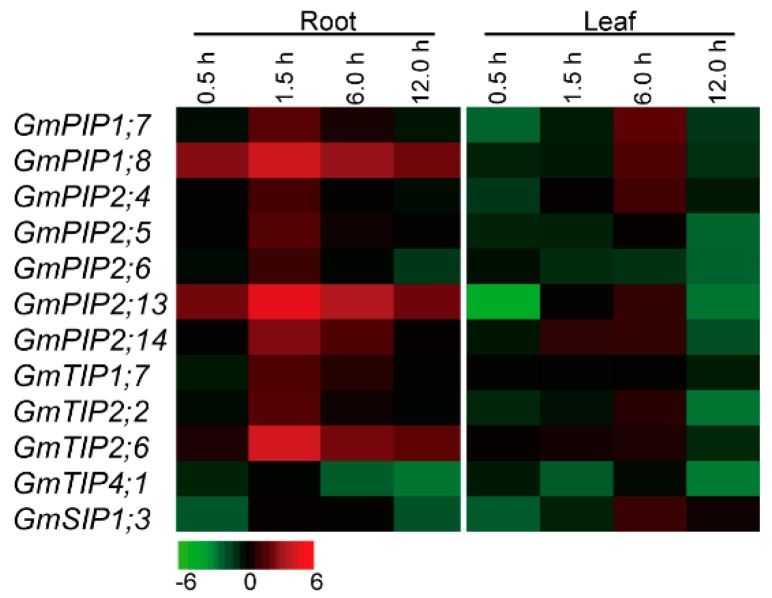
Expression profiles of 12 candidate soybean *AQP* genes under heat stress treatment. 0.5, 1.5, 6.0 and 12 hour represent the treatment times. The color scales represent relative expression data.

To explore the roles of soybean *AQP* genes in response to heat stress, expression profiles of 12 candidate *GmAQPs* were selected for investigation using qRT-PCR, due to relatively extensive expression in various tissues or high expression levels in root or leaf (Figure 3). In two different tissues, heat stress obviously up-regulated the expression of *GmAQPs* during the early durations (1.5 hour) in roots, whereas heat stress slightly up-regulated expression of *GmAQPs* during the late durations (6.0 hour) in leaves (Figure 4; Table S1). In roots, most of the analyzed members (*GmPIP1;7*, *GmPIP1;8*, *GmPIP2;4*, *GmPIP2;5*, *GmPIP2;13*, *GmPIP2;14*, *GmTIP1;7*, *GmTIP2;2*, and *GmTIP2;6*) were transcriptionally up-regulated, whereas *GmTIP4;1* and *GmSIP1;3* were extremely down-regulated after heat treatment. In leaves, the transcripts of *GmPIP2;5*, *GmPIP2;6*, *GmTIP1;7*, and *GmTIP4;1* were inhibited by heat stress, while *GmPIP1;7*, *GmPIP1;8*, *GmPIP2;4*, *GmPIP2;13*, *GmPIP2;14*, *GmTIP2;2*, and *GmSIP1;3* were firstly inhibited and then promoted. Amongst them, *GmPIP1;8*, *GmPIP2;13*, *GmPIP2;14*, and *GmTIP2;6* were relatively dramatically induced in roots, but *GmTIP4;1* was sharply repressed in both the roots and leaves under heat stress.

### 2.7. Expression Profiles of the Candidate AQP Genes in Response to ABA, ACC, SA, and MeJA Signals 

**Figure 5:**
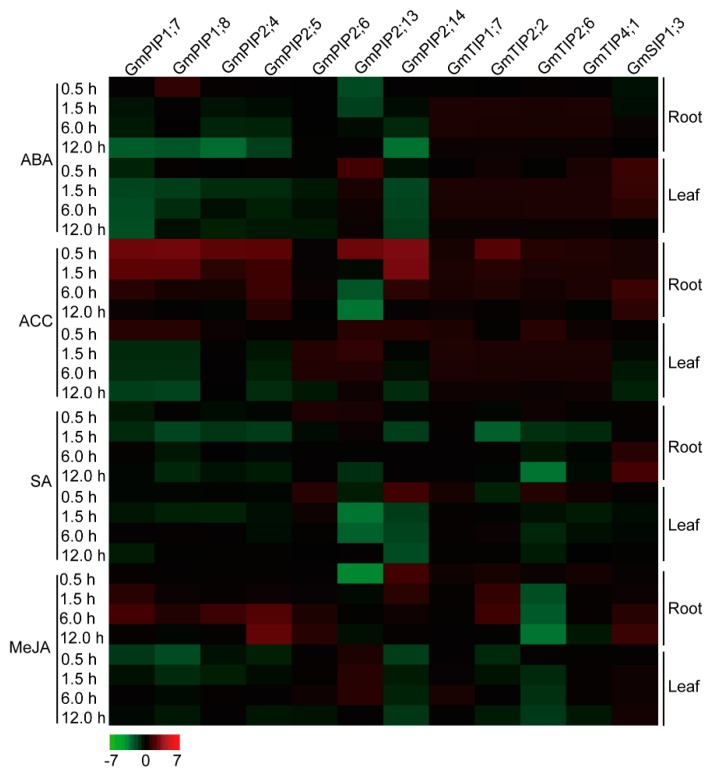
Expression profiles of 12 candidate soybean *AQP* genes under abscisic acid (ABA), l-aminocyclopropane-l-carboxylic acid (ACC), salicylic acid (SA), and methyl jasmonate (MeJA) hormone treatments. 0.5, 1.5, 6.0 and 12 hour represent the treatment times. The color scales represent relative expression data.

Further, these candidate soybean *AQP* genes were subjected to qRT-PCR analyses to evaluate their roles in response to hormone signals (Figure 5; Table S1). For ABA treatment, most transcripts of *PIP* genes underwent down-regulation after ABA treatment, except *GmPIP1;8* and *GmPIP2;13*, whereas *TIP* genes shared up-regulation in both the roots and leaves. *GmPIP1;8* was strongly up-regulated after 0.5 hour of ABA stress in roots and down-regulated in leaves. *GmPIP2;13* was abundantly down-regulated after 0.5 hour of ABA stress in roots and up-regulated in leaves. For ACC treatment, most transcripts of *PIP* genes showed an increase in roots after 0.5 hour, 1.5 hour, and 6.0 hour of ACC stress, except *GmPIP2;13*. However, in leaves, a subset of *PIP* genes (*GmPIP1;7*, *GmPIP1;8*, *GmPIP2;4*, *GmPIP2;5*, *GmPIP2;6*, and *GmPIP2;14*) initially displayed up-regulation expression following gradual down-regulation expression. *GmPIP2;13* was up-regulated only during early duration (0.5 hour) and then down-regulated in roots and continuously up-regulated in leaves. For *TIP* genes, all of them exhibited up-regulated expression after ACC treatment. *GmSIP1;3* was up-regulated in roots and down-regulated in leaves. For SA treatment, a cluster of *PIP* genes (*GmPIP1;7*, *GmPIP1;8*, *GmPIP2;4*, and *GmPIP2;5*) presented down-regulation in both the roots and leaves. *GmPIP2;6* was up-regulated in both the roots and leaves during early durations (0.5 and 1.5 hour) following gradual down-regulation. *GmPIP2;13* was up-regulated after 0.5 hour of SA stress in roots and down-regulated in leaves. *GmPIP2;14* displayed obvious down-regulation in roots and up-regulation in leaves after 0.5 hour of SA stress following down-regulation. For *TIP* genes, the transcriptional level of *GmTIP1;7* was detected with up-regulation in both the roots and leaves. *GmTIP2;2* showed sharp down-regulation after 1.5 hour of SA stress in the roots. *GmTIP4;1* was up-regulated at 0.5 hour of SA stress and then down-regulated in both the roots and leaves. *GmSIP1;3* displayed high transcript abundance in roots and low transcript abundance in leaves. For MeJA treatment, the expression levels of *PIP* genes (*GmPIP1;7*, *GmPIP1;8*, *GmPIP2;4*, *GmPIP2;5*, and *GmPIP2;14*) were promoted in roots and inhibited in leaves, while *GmPIP2;13* was inhibited in roots and promoted in leaves. For *TIP* genes, *GmTIP1;7* maintained up-regulation in both the roots and leaves. *GmTIP2;2* showed up-regulation in roots and down-regulation in leaves. *GmTIP4;1* presented up-regulation during early duration (0.5 hour) following gradual down-regulation in both the roots and leaves. The transcript of *GmSIP1;3* maintained a high level in both the roots and leaves.

### 2.8. Promoter Regulatory Elements of the Candidate AQP Genes

**Figure 6:**
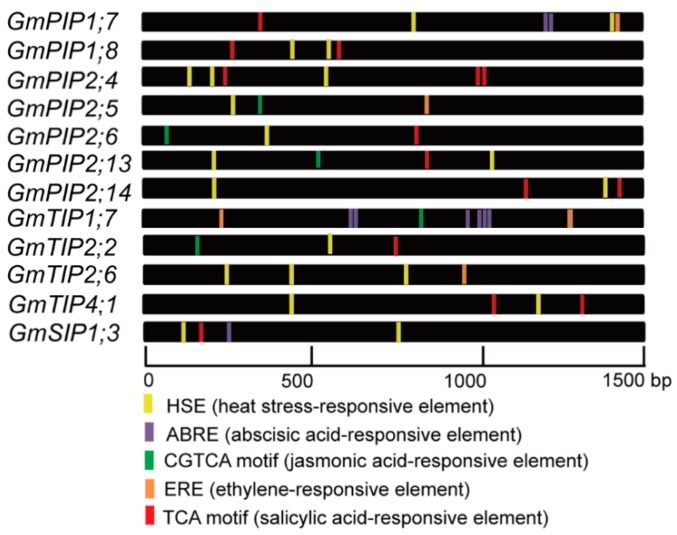
Distribution of *cis*-acting elements in 12 candidate soybean *AQP* gene promoters. 1500 bp adjacent to the *AQP* coding sequence. The elements are represented by different colors. The scale bar represents 500 bp.

1.5 kb sequences, upstream of these 12-candidate soybean *AQP* coding sequences, were analyzed. The *cis*-acting regulatory elements were classified into two types: heat stress and hormone responsive elements (Figure 6; Table 3). Eleven *GmAQPs* were detected having heat stress-related elements. For instance, three HSE elements for *GmPIP2;4* and *GmTIP2;6*; two HSE elements for *GmPIP1;7*, *GmPIP1;8*, *GmTIP4;1*, and *GmSIP1;3* and one HSE element for *GmPIP2;5* and *GmTIP2;2*. Moreover, 3 *GmAQPs* contained ABRE (response to ABA), such as six ABRE elements for *GmTIP1;7*; two ABRE elements for *GmPIP1;7* and one ABRE element for *GmSIP1;3*. Five *GmAQPs* possessed CGTCA (response to MeJA), including one MeJA element for *GmPIP2;5*, *GmPIP2;6*, *GmPIP2;13*, *GmTIP1;7*, and *GmTIP2;2.* 4 *GmAQPs* harbored ERE (response to ethylene), such as two ERE elements for *GmTIP1;7* and one ERE element for *GmPIP1;7*, *GmPIP2;5*, and *GmTIP2;6*. Nine *GmAQPs* contained TCA (response to SA), such as three TCA elements for *GmPIP2;4* and two TCA elements for *GmPIP1;8*, *GmPIP2;14*, and *GmTIP4;1* and two TCA elements for *GmPIP1;7*, *GmPIP2;6*, *GmPIP2;13*, *GmTIP2;2*, and *GmSIP1;3*. Among them, more than one hormone responsive element was observed in the promoter regions of *GmPIP1;7*, *GmPIP2;5*, *GmPIP2;6*, *GmPIP2;13*, *GmTIP1;7*, *GmTIP2;2*, and *GmSIP1;3*.

### 2.9. GUS Activity of the GmTIP2;6 Promoter

**Figure 7:**
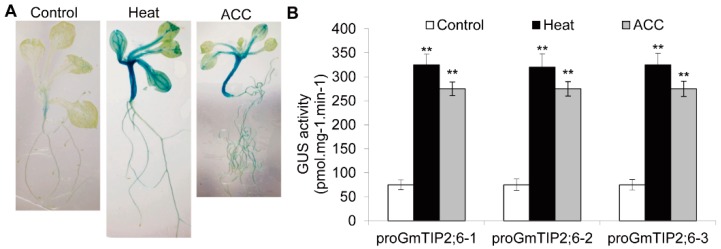
Beta-glucuronidase (GUS) activities of *GmTIP2;6* promoter under heat stress and hormone treatments. (**A**) Transgenic seedlings treated with heat and ACC stress. (**B**) Activity analyses of GUS protein in three proGmTIP2;6-GUS transgenic *Arabidopsis* plants under different treatments. ** indicates significant differences in comparison with the control treatment at *p* < 0.01 (*t*-test).

To characterize the function of *AQP* promoter in response to heat stress and hormone signals, the *GmTIP2;6* promoter, with a relatively high number of HSE elements, was fused to the *GUS* reporter gene and transferred into *Arabidopsis* (Dataset S3). Under normal growth conditions, the expression pattern of the *GUS* gene driven by the *GmTIP2;6* promoter was weakly detected in the hypocotyls (Figure 7A). After heat treatment, GUS activity was remarkably induced and increased in hypocotyls, roots, leaf vascular bundles, and young leaf trichomes. Similarly, after ACC treatment, the responsiveness of the *GmTIP2;6* promoter was enhanced in hypocotyls, roots, leaf vascular bundles, and young leaf trichomes (Figure 7A). Further quantitative GUS activity analyses verified that the translational levels of GUS protein in the transgenic plants with heat and ACC treatments were evidently stronger than those without treatments (Figure 7B). This result confirmed that *GmTIP2;6* was one heat stress and ACC hormone inducible promoter.

## 3. Discussion

AQPs, as representative trans-membrane transporters, have important functions in modulating plant stress tolerance [9,10]. From the recently-updated soybean genome database Phytozome V12.1, 75 putative GmAQPs were identified based on HMM profile, KEGG orthology, BlastP, and BlastN searches (Table 1). All GmAQP proteins possessed six conserved TM helices (TM1 to TM6). Divergent ar/R selectivity filters and FPs were also identified, which were essential for transport specificity of GmAQPs (Table 2). Point mutations or sequence variations of these amino acid residues could confer different substrate permeability in different GmAQP members [30,31,32]. Distinct phosphorylation sites (Ser, Thr, and Tyr) were also detected which might be involved in post-translational modifications of GmAQPs (Table 1). Various environmental conditions such as drought, salinity, or oxidative stresses could induce quantitative changes in PIP, TIP, or NIP phosphorylation at multiple sites on the N-terminal or C-terminal tail [33,34,35]. However, knowledge of the protein kinases and protein phosphatases determining aquaporin phosphorylation is still scarce. All these diverse structure characteristics may allow complex regulation modes for AQPs in response to multiple environmental and hormonal stimuli. Evolutionary analyses showed that *GmAQPs* were categorized into five distinct sub-families (Figure 2). Furthermore, all identified *GmAQPs* were phylogenetically compared to the orthologs derived from the dicotyledonous model plant *Arabidopsis*, which might share highly conservative functions. As *AtPIP2;7* was involved in salinity-mediated transcriptional and post-translational regulation [36], it will be interesting to investigate whether their homologous genes *GmPIP2;1* and *GmPIP2;2* respond to salt stress. AtTIP1;3 was a pollen-specific AQP for transporting water and urea [37], and it will be interesting to test their homologous genes (*GmTIP1;1*, *GmTIP1;2*, *GmTIP1;3*, and *GmTIP1;10*) for similar permeability. *AtTIP5;1* improved plant tolerance to boron toxicity [38], and its homologous gene *GmTIP5;1* might also confer resistance to boron. *AtNIP7;1* enhanced tolerance to arsenate toxicity [39], and its homologous genes (*GmNIP7;1* and *GmNIP7;2*) might also contribute to arsenate stress. Compared with previous report [28], 17 GmAQP proteins were newly identified which were distributed in PIP, TIP, NIP and XIP sub-families: 11 GmPIPs (GmPIP1;9, GmPIP1;10, GmPIP2;3, GmPIP2;4, GmPIP2;8, GmPIP2;9, GmPIP2;10, GmPIP2;11, GmPIP2;12, GmPIP2;13, and GmPIP2;14), 1 GmTIP (GmTIP1;10), 4 GmNIPs (GmNIP1;6, GmNIP3;1, GmNIP4;1, and GmNIP4;2) and 1 GmXIP (GmXIP1;2). Compared with previous report [29], 2 GmPIPs (GmPIP1;9 and GmPIP1;10) and 1 GmTIP (GmTIP1;10) were newly identified. All these newly identified GmAQPs contained the typical and conserved AQP domains as shown in Figures S1–S6. They allow us to re-identify the gene numbers of soybean AQPs on the recently-updated public soybean genome database Phytozome V12.1. Our current detailed analyses will add more potentially functional AQPs to the set of soybean.

Time, location, and level of gene transcripts reflected the functions of *AQPs* under both favorable and stressful conditions [40,41,42]. In the present study, tissue expression patterns of *GmAQPs* were analyzed based on RNA-seq data from the public soybean database. Some transcripts of *GmAQPs* were expressed at high levels while others were expressed at low levels (Figure 3). Most *GmAQPs* extensively functioned in multiple tissues, and individual *GmAQPs* seemed to function in specific tissue, which was highly similar to *AQPs* in other plant species [11,12,13,14,15,16]. Some *AQP* gene pairs at the same evolutionary clade preferred to express in the same tissue. For instance, *GmPIP2;4*, *GmPIP2;5*, *GmPIP2;6,* and *AtPIP2;4*; *GmTIP2;1*, *GmTIP2;2*, and *AtTIP2;2*; and *GmTIP4;1*, *GmTIP4;2*, and *AtTIP4;1* shared high transcript levels in roots. *GmTIP3;1*, *GmTIP3;2*, *GmTIP3;3*, *GmTIP3;4*, and *AtTIP3;1* and *AtTIP3;2* shared seed-specific expression. These results imply that soybean *AQPs* may function in a wide range of developmental processes.

Heat stress modulated the transcription of plant *AQPs*, which opened a new avenue of research for identifying soybean *AQPs* involved in thermo-tolerance [18,19,20,21,22,23]. However, the relationship between *GmAQPs* and heat resistance in soybean still remains elusive. Preliminary heat stress-related element analyses suggested that *GmAQPs* might be involved in modulating plant stress tolerance against heat stimuli (Figure S7). The number of heat stress-related elements in *GmAQP* promoters ranged from 0 to 5. Among them, 11 *GmAQPs* (*GmPIP1;1*, *GmPIP1;4*, *GmPIP2;3*, *GmPIP2;4*, *GmPIP2;12*, *GmTIP1;4*, *GmTIP2;6*, *GmNIP4;1*, *GmNIP5;2*, *GmNIP7;2*, and *GmSIP1;6*) contained more than three heat stress responsive elements. Further expression analyses also confirmed that heat stress could significantly activate or inhibit the expression of candidate *GmAQPs* (Figure 4). Among them, eight *GmAQPs* (*GmPIP1;7*, *GmPIP1;8*, *GmPIP2;4*, *GmPIP2;5*, *GmPIP2;13*, *GmPIP2;14*, *GmTIP1;7*, and *GmTIP2;2*) were favorably accumulated in roots under heat stress. Based on the public database of *Arabidopsis* eFP Browser [16,43], systematical microarray analyses also showed that most *AtAQPs* were involved in the heat stress process (Figure S8; Table S1). All these results indicate that plant *AQPs* serve as targets for modulating thermo-tolerance.

Plant hormones were important signal molecules that controlled plant growth and development in response to heat stimulus, including ABA, SA, and MeJA [44]. However, evidence for the molecular mechanism of *AQPs*’ involvement in the hormone response process remained scanty. The preliminary promoter element analyses indicated that different members of soybean *GmAQPs* possessed distinct hormone-related elements (Figure S7). For instance, two *GmAQPs* (*GmPIP1;1* and *GmNIP4;2*), three *GmAQPs* (*GmPIP2;9*, *GmTIP2;6*, and *GmNIP1;2*), eight *GmAQPs* (*GmPIP1;8*, *GmTIP2;7*, *GmTIP4;1*, *GmNIP1;1*, *GmNIP1;6*, *GmNIP6;3*, *GmSIP1;1*, and *GmSIP2;1*) and three *GmAQPs* (*GmTIP5;1*, *GmNIP7;1*, and *GmSIP1;4*) contained ABA, ET, SA, or MeJA-special element, respectively. In contrast, a combination of four hormone-related elements were observed in *GmPIP1;10*, *GmPIP2;1*, and *GmTIP4;2*. Furthermore, gene expression analyses showed that different *GmAQPs* displayed distinct transcriptional changes, up-regulation or down-regulation under different hormone treatments, as evidenced by the qRT-PCR assay (Figure 5). For example, five highly up-regulated *GmPIPs* transcripts during ACC and MeJA hormone treatments in roots included *GmPIP1;7*, *GmPIP1;8*, *GmPIP2;4*, *GmPIP2;5*, and *GmPIP2;14*, and four abundantly accumulated *GmTIPs* during ABA and ACC hormone treatments in both the roots and leaves included *GmTIP1;7*, *GmTIP2;2*, *GmTIP2;6*, and *GmTIP4;1*. Moreover, the expression of *GmSIP1;3* got enhanced and underwent significant changes during ACC, SA, and MeJA hormone treatments in roots. However, the transcript changes of four *GmPIPs* (*GmPIP1;7*, *GmPIP1;8*, *GmPIP2;4*, and *GmPIP2;5*) were observed with down-regulation under ABA, SA, and MeJA hormone treatments in leaves. In rice and oilseed rape, ABA highly enhanced the expression of *OsTIP1;1* in the shoots and roots and *BnTIP2* in the seeds [45,46]. In resurrection plant, ABA greatly decreased the expression of *CpTIP* in the callus [47]. In wheat, ethylene (ET) up-regulated the gene expression of wheat *TaAQP8* (one *PIP* sub-family gene) under salt stress [48]. In rose, ET decreased the expression of *RhTIP1;1* in the flower [49]. Systematical microarray analysis of *AQPs* under ABA, ACC, and MeJA based on the public database of *Arabidopsis* eFP Browser showed that most *Arabidopsis AQPs* were involved in the process (Figure S9; Table S1) [16,43]. Other stress-related signals such as brassinolide (BR), gibberellin (GA), and auxin (IAA) also regulated the expression of *AQPs* [50,51,52,53] but by, as yet, unclear mechanisms. All this evidence gives clues to the role of *AQPs* in complicated hormone signal transduction systems in different tissues. In this regard, it will be of interest to decipher further how *AQP* genes work in the interactive signaling regulation network, especially under heat stress.

Promoters, the direct indication of gene expression patterns, were extensively involved in the responses of signal molecules and environmental elicitors. It was noteworthy that we validated that soybean *GmTIP2;6* promoter was a typical inducible promoter, which responded to ACC and heat stresses in hypocotyls, vascular bundles, and leaf trichomes (Figure 7). This was consistent with the qRT-PCR result that *GmTIP2;6* was up-regulated by heat stress and ACC hormone (Figure 4 and Figure 5). In cotton, strong expression of the *GUS* gene driven by *GhPIP2;7* promoter was detected in leaves of 5 to 10-day-old transgenic *Arabidopsis* seedlings, but GUS activity gradually became weak as the seedlings further developed. GUS activity driven by cotton *GhPIP2;7* promoter was remarkably increased after mannitol treatment [54]. In *Arabidopsis*, *AtNIP3;1* promoter-mediated GUS activity was specifically expressed in the roots [55]. The expression of the *GUS* gene driven by *AtPIP2;7* promoter was strongly detected in cotyledons, emerging leaf primordia, and root elongation zones, and salt stress induced strong repression of *AtPIP2;7* promoter activity [35]. In soybean, the GUS activity of *GmTIP2;3* promoter was expressed in the root, stem, and leaf and preferentially expressed in the stele of root and stem [56]. These data indicated different promoters of *AQP* members played different roles in different tissues or development stages. In the continued study, it will be very meaningful to investigate the core elements of *AQP* promoters for quantitative and qualitative gene expression regulation.

## 4. Materials and Methods

### 4.1. Categorization of Soybean AQP Genes

The soybean genome sequences were retrieved from Phytozome V12.1 (http://phytozome.jgi.doe.gov/pz/portal.html). The keyword searches of aquaporin, Hidden Markov model profile (PF00230), and KEGG Orthology terms (PIPs, K09872; TIPs, K09873; NIPs, K09874; SIPs, K09875) were applied to identify the soybean candidate AQP members [57]. Known *Arabidopsis*, *Zea mays*, *Oryza sativa*, *Populus trichocarapa*, *Phaseolus vulgaris* and *Lotus japonicas* AQPs were also subjected to BlastP and BlastN against the soybean database with cut-off *E*-value of e^−5^ [58,59,60,61]. GmAQPs were named based on their sequence homology with known AQPs and soybean genome annotation. The decrease redundancy tool (http://web.expasy.org/decrease_redundancy/) was utilized to discard the redundant AQP sequences. Further, the resulting candidate sequences were checked for the presence of six TM domains and two NPA motifs by SMART (http://smart.embl-heidelberg.de/smart/batch.pl) and NCBI-CDD (http://www.ncbi.nlm.nih.gov/Structure/cdd/wrpsb.cgi) web servers. The numbers of phosphorylation sites of AQP proteins were predicted with NetPhos 2.0 (http://www.cbs.dtu.dk/services/NetPhos/). The molecular weight (MW) and isoelectric point (*pI*) of AQP proteins were calculated using ExPASy (http://web.expasy.org). The chromosomal positions of *GmAQPs* were mapped using MapInspect software based on the starting position of all genes on each chromosome. Tandem duplications were identified manually. Adjacent genes of the same sub-group tightly linked within 20 kb of each other and the identity of the genes ≥80% are considered as tandem duplicated genes [62].

### 4.2. Phylogenetic Tree

The GmAQP full length protein sequences were aligned using ClustalX2 software. As soon as the ALN file was generated, MEGA6 was carried out to construct the neighbor-joining (NJ) phylogenetic tree [63]. The criteria were adopted with pairwise deletion option and Poisson correction model. Bootstrap test was performed with 1000 replicates.

### 4.3. Tissular Expression Profile Analyses

The RNA-seq data of *GmAQP* genes in different tissues, including leaf, stem, root, flower, seed, root hair, pod, SAM, and nodule, was available from Phytozome V12.1 database [64]. BAR HeatMapper Tool (http://bar.utoronto.ca/ntools/cgi-bin/ntools_heatmapper.cgi) was carried out to display the expression profiles of *AQP* genes in heatmaps [16].

### 4.4. Heat Stress and Hormone Treatments

Soybean cultivar GMLN012012017, with the characteristic of heat tolerance, was used in this study. Soybean seeds were cultivated in pots in an illuminated incubator (PTC-300, Shanghai, China) adjusted to 22 °C temperature, 60% relative humidity, 16/8 h photoperiod, and25000 Lux light intensity. For high-temperature treatment, 21-day-old seedlings in pots were transferred to the illuminated incubator adjusted to 42 °C. The un-treated samples were used as the control (0 hour). For hormone treatments, the root systems of 21-day-old seedlings were washed gently with water to remove soil and then the plants were soaked into 200 mL solutions with 100 µM abscisic acid (ABA), 100 µM l-aminocyclopropane-l-carboxylic acid (ACC), 100 µM salicylic acid (SA) or 100 µM methyl jasmonate (MeJA). The samples soaked with water were used as the control (0 hour). Each single seedling was sampled at one time point (0, 0.5, 1.5, 6.0 or 12 hour). Then, whole leaves and roots of each control or treated seedling were collected and frozen in liquid nitrogen and stored at −80 °C for further analyses.

### 4.5. qRT-PCR

Total RNA was separately extracted from the frozen samples using a RNA Simple Total RNA Extraction Kit (Tiangen, Beijing, China) according to the manufacturer’s protocol. Then, the cDNA was synthesized using a FastQuant RT Kit (Tiangen, Beijing, China). Gene-specific primers were designed using PrimerQuest Tool (http://sg.idtdna.com/PrimerQuest/Home/) (Table S2). *GmActin11* (Glyma.18G290800) was selected as the internal reference gene [65,66,67,68]. The amplification reactions were performed on Applied Biosystems StepOnePlus^TM^ Real-Time System using KAPA SYBR^®^Fast qPCR Kit (Tiangen, Beijing, China) with the following parameters: initializing denaturation at 95 °C for 5 min, followed by 45 cycles of denaturation at 95 °C for 5 seconds, annealing at 58 °C for 5 seconds, and extension at 72 °C for 30 seconds. Three technical replicates were maintained for each sample. The relative expression levels were calculated as 2^−ΔΔ*C*t^ [69]. The heatmaps for the expression profiles of *GmAQP* genes were generated with BAR HeatMapper Tool [16].

### 4.6. Promoter Element Prediction

1.5 kb promoter regions, upstream of the *AQP* gene coding sequences, were extracted from Phytozome V12.1. Promoter regions were subsequently analyzed using PlantCARE database (http://bioinformatics.psb.ugent.be/webtools/plantcare/html/) to illustrate the number and composition of hormone and heat stress responsive elements.

### 4.7. Promoter Cloning and Arabidopsis Transformation

The promoter of *GmTIP2;6* was isolated using the specific primer pairs (Table S2). To generate the proGmTIP2;6::GUS construct, the *CaMV 35S* promoter was replaced by the promoter of *GmTIP2;6*. The promoter of *GmTIP2;6* was inserted into *Sal* I/*Sma* I sites and sub-cloned into the pBI121 vector whose *Hind* III site was replaced by three continuous sites (*Hind* III, *Pst* I, and *Sal* I) upstream of the *CaMV 35S* promoter (Figure S10). The GUS fusion construct was then introduced into *Arabidopsis* (Col-0) by *Agrobacterium*-mediated floral-dip method [70,71]. Transformed seeds were selected on MS medium with 50 mg/L kanamycin (Kan). Homozygous lines of T3 were used for the following GUS activity assays.

### 4.8. GUS Activity Detection

To evaluate GUS activity, the proGmTIP2;6::GUS transgenic seeds were sowed on MS medium for 5 days, and then transferred to MS medium supplemented with 100 µM ACC for 7 days. For the high-temperature treatment, 5-day-old seedlings on MS medium were transferred to the temperature-controlled chamber adjusted to 37 °C for 7 days. Seedlings on MS medium without any additions were used as controls [72]. GUS staining was conducted using standard protocols. In brief, seedlings were incubated in 10 mL tubes with 1 mg/mL 5-bromo-4-chloro-3-indolylglucuronic acid, 5 mM potassium ferricyanide, 5 mM potassium ferrocyanide, 0.03% Triton X-100, and 0.1 M sodium phosphate buffer, pH 7.0 overnight at 37 °C. Then, seedlings were immersed in 70% ethanol to remove the chlorophyll and visualized on the Leica microscope. GUS activities were measured by monitoring the cleavage of GUS substrate 4-methylumbelliferyl glucuronide as reported previously [73]. Data analyses of variance were used to compare the statistical difference based on Student’s *t*-test, at a significant level of *p* < 0.01.

## Figures and Tables

**Table 1:** Nomenclature of *aquaporin* (*AQP*) genes in soybean.

Gene Number	Gene Name	Gene Symbol	Chromosome Location	CDS Length (bp)	Protein Length (aa)	*pI*	MW (kDa)	Numbers of Phosphorylation Sites
*GmAQP1*	*GmPIP1;1*	Glyma.03G078700	3:18018230..18021565	855	284	9.10	30.41	Ser: 7 Thr: 1 Tyr: 2
*GmAQP2*	*GmPIP1;2*	Glyma.18G198300	18:51879812..51881980	864	287	9.26	30.89	Ser: 8 Thr: 3 Tyr: 2
*GmAQP3*	*GmPIP1;3*	Glyma.01G220600	1:54066066..54068057	861	286	9.13	30.79	Ser: 7 Thr: 1 Tyr: 1
*GmAQP4*	*GmPIP1;4*	Glyma.11G023200	11:1656129..1658174	861	286	8.84	30.74	Ser: 6 Thr: 1 Tyr: 1
*GmAQP5*	*GmPIP1;5*	Glyma.05G208700	5:41267148..41268807	864	287	9.00	30.89	Ser: 9 Thr: 1 Tyr: 2
*GmAQP6*	*GmPIP1;6*	Glyma.08G015300	8:1202356..1204135	870	289	8.61	30.89	Ser: 9 Thr: 1 Tyr: 2
*GmAQP7*	*GmPIP1;7*	Glyma.14G061500	14:4894197..4896207	870	289	8.60	30.61	Ser: 9 Thr: 3 Tyr: 3
*GmAQP8*	*GmPIP1;8*	Glyma.11G228000	11:36767510..36769078	870	289	7.01	30.77	Ser: 11 Thr: 2 Tyr: 3
*GmAQP9*	*GmPIP1;9*	Glyma.01G113400	1:1138834287..38837846	1092	363	9.31	39.41	Ser: 14 Thr: 11 Tyr: 7
*GmAQP10*	*GmPIP1;10*	Glyma.02G255000	2:44207467..44209844	960	319	9.38	34.19	Ser: 12 Thr: 8 Tyr: 4
*GmAQP11*	*GmPIP2;1*	Glyma.04G003200	4:227991..229365	828	275	9.45	29.30	Ser: 9 Thr: 0 Tyr: 2
*GmAQP12*	*GmPIP2;2*	Glyma.06G003200	6:264336..265850	837	278	9.35	29.30	Ser: 9 Thr: 0 Tyr: 2
*GmAQP13*	*GmPIP2;3*	Glyma.11G146500	11:11300751..11303007	861	286	6.95	29.55	Ser: 8 Thr: 7 Tyr: 4
*GmAQP14*	*GmPIP2;4*	Glyma.12G075400	12:5747587..5750039	861	286	6.19	30.38	Ser: 7 Thr: 9 Tyr: 3
*GmAQP15*	*GmPIP2;5*	Glyma.12G172500	12:32929324..32931027	864	287	8.25	30.67	Ser: 9 Thr: 2 Tyr: 4
*GmAQP16*	*GmPIP2;6*	Glyma.13G325900	13:40664607..40666361	864	287	8.26	30.84	Ser: 8 Thr: 2 Tyr: 3
*GmAQP17*	*GmPIP2;7*	Glyma.03G180900	3:41279731..41281496	861	286	8.98	30.79	Ser: 12 Thr: 8 Tyr: 4
*GmAQP18*	*GmPIP2;8*	Glyma.19G181300	19:44007407..44009765	858	285	9.15	30.61	Ser: 13 Thr: 9 Tyr: 4
*GmAQP19*	*GmPIP2;9*	Glyma.02G073600	2:6421649..6424849	858	285	8.29	30.56	Ser: 10 Thr: 6 Tyr: 1
*GmAQP20*	*GmPIP2;10*	Glyma.16G155000	16:31513389..31517035	858	285	8.29	30.68	Ser: 10 Thr: 6 Tyr: 3
*GmAQP21*	*GmPIP2;11*	Glyma.16G155100	16:31522994..31524889	858	285	8.29	30.41	Ser: 10 Thr: 6 Tyr: 3
*GmAQP22*	*GmPIP2;12*	Glyma.02G073700	2:6434383..6437873	858	285	8.59	30.37	Ser: 11 Thr: 6 Tyr: 3
*GmAQP23*	*GmPIP2;13*	Glyma.10G211000	10:44343751..44346957	891	296	7.70	30.44	Ser: 9 Thr: 6 Tyr: 3
*GmAQP24*	*GmPIP2;14*	Glyma.20G179700	20:41738693..41741581	855	284	8.29	31.73	Ser: 10 Thr: 7 Tyr: 4
*GmAQP25*	*GmTIP1;1*	Glyma.02G094700	2:8409966..8411440	759	252	5.12	25.96	Ser: 3 Thr: 3 Tyr: 0
*GmAQP26*	*GmTIP1;2*	Glyma.18G286700	18:60989768..60991401	759	252	5.49	26.04	Ser: 4 Thr: 2 Tyr: 0
*GmAQP27*	*GmTIP1;3*	Glyma.10G290600	10:50271428..50272965	759	252	6.01	26.02	Ser: 3 Thr: 2 Tyr: 0
*GmAQP28*	*GmTIP1;4*	Glyma.11G143100	11:10892421..10894109	759	252	5.37	25.79	Ser: 5 Thr: 1 Tyr: 1
*GmAQP29*	*GmTIP1;5*	Glyma.12G066200	12:4870480..4871652	738	245	6.02	25.03	Ser: 5 Thr: 1 Tyr: 1
*GmAQP30*	*GmTIP1;6*	Glyma.13G333100	13:41270585..41271998	759	252	5.16	26.01	Ser: 2 Thr: 1 Tyr: 0
*GmAQP31*	*GmTIP1;7*	Glyma.03G185900	3:41779243..41780564	753	250	6.01	25.45	Ser: 0 Thr: 3 Tyr: 1
*GmAQP32*	*GmTIP1;8*	Glyma.19G186100	19:44258426..44259853	753	250	6.01	25.53	Ser: 0 Thr: 3 Tyr: 1
*GmAQP33*	*GmTIP1;9*	Glyma.13G146300	13:24436182..24438466	753	250	10.01	26.54	Ser: 8 Thr: 2 Tyr: 0
*GmAQP34*	*GmTIP1;10*	Glyma.20G098600	20:34184591..34191923	732	243	6.17	25.52	Ser: 6 Thr: 3 Tyr: 0
*GmAQP35*	*GmTIP2;1*	Glyma.01G208200	1:53110677..53113455	750	249	5.08	25.28	Ser: 4 Thr: 2 Tyr: 1
*GmAQP36*	*GmTIP2;2*	Glyma.11G034000	11:2476012..2478825	750	249	5.08	25.32	Ser: 2 Thr: 1 Tyr: 2
*GmAQP37*	*GmTIP2;3*	Glyma.07G018000	7:1435523..1437651	747	248	5.69	25.23	Ser: 6 Thr: 1 Tyr: 1
*GmAQP38*	*GmTIP2;4*	Glyma.08G203000	8:16535219..16537122	747	248	5.69	25.27	Ser: 3 Thr: 2 Tyr: 2
*GmAQP39*	*GmTIP2;5*	Glyma.13G356000	13:43018922..43020336	744	247	5.51	25.07	Ser: 2 Thr: 2 Tyr: 2
*GmAQP40*	*GmTIP2;6*	Glyma.15G018100	15:1393557..1395809	744	247	5.50	25.07	Ser: 3 Thr: 3 Tyr: 1
*GmAQP41*	*GmTIP2;7*	Glyma.19G035400	19:4625496..4626575	714	237	5.57	24.08	Ser: 4 Thr: 1 Tyr: 1
*GmAQP42*	*GmTIP3;1*	Glyma.09G160500	9:35913523..35915582	768	255	6.54	27.03	Ser: 5 Thr: 1 Tyr: 1
*GmAQP43*	*GmTIP3;2*	Glyma.16G210000	16:36421819..36424304	768	255	6.54	27.11	Ser: 5 Thr: 0 Tyr: 1
*GmAQP44*	*GmTIP3;3*	Glyma.10G174400	10:40238530..40240337	765	254	7.13	27.08	Ser: 5 Thr: 0 Tyr: 0
*GmAQP45*	*GmTIP3;4*	Glyma.20G216100	20:44068541..44070258	765	254	7.88	27.07	Ser: 5 Thr: 3 Tyr: 1
*GmAQP46*	*GmTIP4;1*	Glyma.04G083200	4:7019276..7020984	741	246	5.71	25.65	Ser: 3 Thr: 2 Tyr: 0
*GmAQP47*	*GmTIP4;2*	Glyma.06G084600	6:6498818..6500103	741	246	5.71	25.63	Ser: 5 Thr: 2 Tyr: 2
*GmAQP48*	*GmTIP5;1*	Glyma.09G224700	9:41742635..41743884	759	252	7.82	26.30	Ser: 9 Thr: 2 Tyr: 2
*GmAQP49*	*GmNIP1;1*	Glyma.05G162600	5:35105884..35108185	813	270	9.67	28.68	Ser: 8 Thr: 5 Tyr: 1
*GmAQP50*	*GmNIP1;2*	Glyma.08G120200	8:9268559..9270946	825	274	9.48	29.27	Ser: 9 Thr: 3 Tyr: 1
*GmAQP51*	*GmNIP1;3*	Glyma.13G224900	13:32551102..32553703	822	273	7.76	28.93	Ser: 6 Thr: 4 Tyr: 2
*GmAQP52*	*GmNIP1;4*	Glyma.15G087300	15:6704209..6706791	822	273	7.74	28.83	Ser: 6 Thr: 4 Tyr: 2
*GmAQP53*	*GmNIP1;5*	Glyma.08G120100	8:9262302..9265834	816	271	6.41	28.91	Ser: 9 Thr: 3 Tyr: 5
*GmAQP54*	*GmNIP1;6*	Glyma.05G162500	5:35371190..35375992	816	271	8.87	28.66	Ser: 11 Thr: 8 Tyr: 1
*GmAQP55*	*GmNIP2;1*	Glyma.07G217700	7:39062920..39065820	789	262	8.14	28.13	Ser: 4 Thr: 2 Tyr: 1
*GmAQP56*	*GmNIP3;1*	Glyma.14G174300	14:43721841..43723560	813	270	8.23	28.66	Ser: 15 Thr: 11 Tyr: 3
*GmAQP57*	*GmNIP4;1*	Glyma.02G246700	2:46541265..46543675	786	261	7.61	27.61	Ser: 23 Thr: 5 Tyr: 4
*GmAQP58*	*GmNIP4;2*	Glyma.14G069500	14:5711153..5714115	786	261	8.25	27.59	Ser: 17 Thr: 4 Tyr: 4
*GmAQP59*	*GmNIP5;1*	Glyma.09G238200	9:42824943..42829709	882	293	8.55	30.44	Ser: 28 Thr: 9 Tyr: 1
*GmAQP60*	*GmNIP5;2*	Glyma.10G221100	10:44670892..44676555	900	299	7.68	31.15	Ser: 6 Thr: 5 Tyr: 0
*GmAQP61*	*GmNIP6;1*	Glyma.18G259500	18:58816436..58821548	888	295	6.96	30.55	Ser: 19 Thr: 5 Tyr: 0
*GmAQP62*	*GmNIP6;2*	Glyma.08G217400	8:17701761..17706495	921	306	9.13	31.74	Ser: 8 Thr: 5 Tyr: 0
*GmAQP63*	*GmNIP6;3*	Glyma.15G003900	15:355676..359967	915	304	8.25	31.28	Ser: 7 Thr: 2 Tyr: 0
*GmAQP64*	*GmNIP7;1*	Glyma.02G140500	2:14348789..14351092	891	296	8.46	31.42	Ser: 10 Thr: 1 Tyr: 3
*GmAQP65*	*GmNIP7;2*	Glyma.10G033600	10:2898411..2900795	870	289	8.69	30.82	Ser: 7 Thr: 1 Tyr: 1
*GmAQP66*	*GmSIP1;1*	Glyma.02G069800	2:6061309..6065568	921	306	9.27	26.64	Ser: 0 Thr: 2 Tyr: 0
*GmAQP67*	*GmSIP1;2*	Glyma.16G151300	16:30813218..30817735	738	245	9.27	26.43	Ser: 0 Thr: 2 Tyr: 0
*GmAQP68*	*GmSIP1;3*	Glyma.19G108400	19:35912781..35923174	747	248	9.12	26.56	Ser: 1 Thr: 2 Tyr: 3
*GmAQP69*	*GmSIP1;4*	Glyma.16G043800	16:4096288..4102424	747	248	9.10	26.52	Ser: 2 Thr: 2 Tyr: 2
*GmAQP70*	*GmSIP1;5*	Glyma.12G097800	12:8369034..8369846	720	239	9.99	26.02	Ser: 4 Thr: 3 Tyr: 1
*GmAQP71*	*GmSIP1;6*	Glyma.06G307000	6:48987251..48988278	720	239	9.91	25.87	Ser: 4 Thr: 4 Tyr: 1
*GmAQP72*	*GmSIP2;1*	Glyma.03G119300	3:35075328..35078322	693	230	9.45	25.24	Ser: 6 Thr: 7 Tyr: 0
*GmAQP73*	*GmSIP2;2*	Glyma.19G123600	19:37949307..37951724	711	236	9.45	25.97	Ser: 10 Thr: 6 Tyr: 0
*GmAQP74*	*GmXIP1;1*	Glyma.12G023600	12:1729006..1730580	939	312	7.02	33.75	Ser: 6 Thr: 2 Tyr: 3
*GmAQP75*	*GmXIP1;2*	Glyma.11G097800	11:7449914..7450765	852	283	6.50	30.12	Ser: 9 Thr: 5 Tyr: 3

**Table 2:** Conserved amino acid residues (Asn-Pro-Ala (NPA) motifs, aromatic/arginine (ar/R) filters and Froger’s positions (FPs)) and trans-membrane (TM) domains of AQP proteins in soybean.

Gene Name	Gene Symbol	TM Number	NPA Motifs		ar/R Selectivity Filters				FPs				
			LB	LE	H2	H5	LE1	LE2	P1	P2	P3	P4	P5
*GmPIP1;1*	Glyma.03G078700	6	NPA	NPA	F	H	T	R	E	S	A	F	W
*GmPIP1;2*	Glyma.18G198300	6	NPA	NPA	F	H	T	R	Q	S	A	F	W
*GmPIP1;3*	Glyma.01G220600	6	NPA	NPA	F	H	T	R	E	S	A	F	W
*GmPIP1;4*	Glyma.11G023200	6	NPA	NPA	F	H	T	R	E	S	A	F	W
*GmPIP1;5*	Glyma.05G208700	6	NPA	NPA	F	H	T	R	E	S	A	F	W
*GmPIP1;6*	Glyma.08G015300	6	NPA	NPA	F	H	T	R	E	S	A	F	W
*GmPIP1;7*	Glyma.14G061500	6	NPA	NPA	F	H	T	R	E	S	A	F	W
*GmPIP1;8*	Glyma.11G228000	6	NPA	NPA	F	H	T	R	E	S	A	F	W
*GmPIP1;9*	Glyma.01G113400	6	NPA	NPA	F	H	T	R	E	S	A	F	W
*GmPIP1;10*	Glyma.02G255000	6	NPA	NPA	F	H	T	R	E	S	A	F	W
*GmPIP2;1*	Glyma.04G003200	6	NPA	NPA	F	H	T	R	M	S	A	F	W
*GmPIP2;2*	Glyma.06G003200	6	NPA	NPA	F	H	T	R	M	S	A	F	W
*GmPIP2;3*	Glyma.11G146500	6	NPA	NPA	F	H	T	R	Q	S	A	F	W
*GmPIP2;4*	Glyma.12G075400	6	NPA	NPA	F	H	T	R	Q	S	A	F	W
*GmPIP2;5*	Glyma.12G172500	6	NPA	NPA	F	H	T	R	Q	S	A	Y	W
*GmPIP2;6*	Glyma.13G325900	6	NPA	NPA	F	H	T	R	Q	S	A	Y	W
*GmPIP2;7*	Glyma.03G180900	6	NPA	NPA	F	H	T	R	Q	S	A	F	W
*GmPIP2;8*	Glyma.19G181300	6	NPA	NPA	F	H	T	R	Q	S	A	F	W
*GmPIP2;9*	Glyma.02G073600	6	NPA	NPA	F	H	T	R	Q	S	A	F	W
*GmPIP2;10*	Glyma.16G155000	6	NPA	NPA	F	H	T	R	Q	S	A	F	W
*GmPIP2;11*	Glyma.16G155100	6	NPA	NPA	F	H	T	R	Q	S	A	F	W
*GmPIP2;12*	Glyma.02G073700	6	NPA	NPA	F	H	T	R	Q	S	A	F	W
*GmPIP2;13*	Glyma.10G211000	6	NPA	NPA	F	H	T	R	Q	S	A	F	W
*GmPIP2;14*	Glyma.20G179700	6	NPA	NPA	F	H	T	R	Q	S	A	F	W
*GmTIP1;1*	Glyma.02G094700	6	NPA	NPA	H	I	A	V	T	S	A	Y	W
*GmTIP1;2*	Glyma.18G286700	6	NPA	NPA	H	I	A	V	T	S	A	Y	W
*GmTIP1;3*	Glyma.10G290600	6	NPA	NPA	H	I	A	V	T	C	A	Y	W
*GmTIP1;4*	Glyma.11G143100	6	NPA	NPA	H	I	A	V	T	S	A	Y	W
*GmTIP1;5*	Glyma.12G066200	6	NPA	NPA	H	I	A	V	T	S	A	Y	W
*GmTIP1;6*	Glyma.13G333100	6	NPA	NPA	H	I	A	V	T	S	A	Y	W
*GmTIP1;7*	Glyma.03G185900	6	NPA	NPA	H	I	A	V	T	T	A	Y	W
*GmTIP1;8*	Glyma.19G186100	6	NPA	NPA	H	I	A	V	T	T	A	Y	W
*GmTIP1;9*	Glyma.13G146300	6	NPA	NPA	H	I	A	A	T	S	A	Y	W
*GmTIP1;10*	Glyma.20G098600	6	NPA	NPA	H	I	A	V	T	S	A	Y	W
*GmTIP2;1*	Glyma.01G208200	6	NPA	NPA	H	I	G	R	T	S	A	Y	W
*GmTIP2;2*	Glyma.11G034000	6	NPA	NPA	H	I	G	R	T	S	A	Y	W
*GmTIP2;3*	Glyma.07G018000	6	NPA	NPA	H	I	G	R	T	S	A	Y	W
*GmTIP2;4*	Glyma.08G203000	6	NPA	NPA	H	I	G	R	T	S	A	Y	W
*GmTIP2;5*	Glyma.13G356000	6	NPA	NPA	H	I	G	R	T	S	A	Y	W
*GmTIP2;6*	Glyma.15G018100	6	NPA	NPA	H	I	G	R	T	S	A	Y	W
*GmTIP2;7*	Glyma.19G035400	6	NPA	NPA	H	I	G	R	T	S	A	Y	W
*GmTIP3;1*	Glyma.09G160500	6	NPA	NPA	H	I	A	L	T	A	S	F	W
*GmTIP3;2*	Glyma.16G210000	6	NPA	NPA	H	I	A	L	T	A	S	F	W
*GmTIP3;3*	Glyma.10G174400	6	NPA	NPA	H	I	A	R	T	A	A	F	W
*GmTIP3;4*	Glyma.20G216100	6	NPA	NPA	H	I	A	R	T	A	A	F	W
*GmTIP4;1*	Glyma.04G083200	6	NPA	NPA	H	I	A	R	S	S	A	Y	W
*GmTIP4;2*	Glyma.06G084600	6	NPA	NPA	H	I	A	R	S	S	A	Y	W
*GmTIP5;1*	Glyma.09G224700	6	NPA	NPA	S	V	G	C	V	A	A	Y	W
*GmNIP1;1*	Glyma.05G162600	6	NPA	NPA	W	V	A	R	F	S	A	Y	V
*GmNIP1;2*	Glyma.08G120200	6	NPA	NPA	W	V	A	R	F	S	A	Y	V
*GmNIP1;3*	Glyma.13G224900	6	NPA	NPA	W	V	A	R	F	S	A	Y	V
*GmNIP1;4*	Glyma.15G087300	6	NPA	NPA	W	V	A	R	F	S	A	Y	V
*GmNIP1;5*	Glyma.08G120100	6	NPA	NPA	W	V	A	R	F	S	A	Y	L
*GmNIP1;6*	Glyma.05G162500	6	NPA	NPV	W	V	A	R	F	S	A	Y	L
*GmNIP2;1*	Glyma.07G217700	6	NPA	NPA	W	V	A	R	F	S	A	Y	V
*GmNIP3;1*	Glyma.14G174300	6	NPA	NPA	S	V	A	R	Y	S	A	Y	I
*GmNIP4;1*	Glyma.02G246700	6	NPA	NPA	W	V	A	R	L	S	A	Y	V
*GmNIP4;2*	Glyma.14G069500	6	NPA	NPA	W	V	A	R	L	S	A	Y	V
*GmNIP5;1*	Glyma.09G238200	6	NPA	NPA	G	S	G	R	L	T	A	Y	F
*GmNIP5;2*	Glyma.10G221100	6	NPS	NPV	A	I	G	R	Y	T	A	Y	L
*GmNIP6;1*	Glyma.18G259500	6	NPA	NPA	G	S	G	R	L	T	A	Y	F
*GmNIP6;2*	Glyma.08G217400	6	NPA	NPV	N	I	S	R	F	T	A	Y	L
*GmNIP6;3*	Glyma.15G003900	6	NPA	NPV	T	I	G	R	Y	T	A	Y	L
*GmNIP7;1*	Glyma.02G140500	6	NPA	NPA	A	V	G	R	Y	S	A	Y	M
*GmNIP7;2*	Glyma.10G033600	6	NPA	NPA	A	V	G	R	Y	S	A	Y	M
*GmSIP1;1*	Glyma.02G069800	6	NPT	NPA	I	I	P	F	M	A	A	Y	W
*GmSIP1;2*	Glyma.16G151300	6	NPT	NPA	I	I	P	F	M	A	A	Y	W
*GmSIP1;3*	Glyma.19G108400	6	NPT	NPA	V	V	P	N	M	A	A	Y	W
*GmSIP1;4*	Glyma.16G043800	6	NPT	NPA	V	V	P	N	M	A	A	Y	W
*GmSIP1;5*	Glyma.12G097800	6	NPS	NPA	N	A	P	N	L	A	A	Y	W
*GmSIP1;6*	Glyma.06G307000	6	NPS	NPA	N	A	P	N	L	A	A	Y	W
*GmSIP2;1*	Glyma.03G119300	6	NP_	NPA	S	H	G	S	I	V	A	Y	W
*GmSIP2;2*	Glyma.19G123600	6	NP_	NPA	S	H	G	S	I	V	A	Y	W
*GmXIP1;1*	Glyma.12G023600	6	NPI	SPA	V	V	A	R	E	C	A	F	W
*GmXIP1;2*	Glyma.11G097800	6	SPV	NPA	V	V	V	R	D	C	A	F	W

**Table 3:** Number of *cis*-acting elements in 12 candidate soybean *AQP* gene promoters.

Gene Name	Gene Symbol	HSE	ABRE	CGTCA	ERE	TCA
*GmPIP1;7*	Glyma.14G061500	2	2	0	1	1
*GmPIP1;8*	Glyma.11G228000	2	0	0	0	2
*GmPIP2;4*	Glyma.12G075400	3	0	0	0	3
*GmPIP2;5*	Glyma.12G172500	1	0	1	1	0
*GmPIP2;6*	Glyma.13G325900	1	0	1	0	1
*GmPIP2;13*	Glyma.10G211000	2	0	1	0	1
*GmPIP2;14*	Glyma.20G179700	2	0	0	0	2
*GmTIP1;7*	Glyma.03G185900	0	6	1	2	0
*GmTIP2;2*	Glyma.11G034000	1	0	1	0	1
*GmTIP2;6*	Glyma.15G018100	3	0	0	1	0
*GmTIP4;1*	Glyma.04G083200	2	0	0	0	2
*GmSIP1;3*	Glyma.19G108400	2	1	0	0	1

## Supplementary Materials

The following are available online at http://www.mdpi.com/1422-0067/20/2/262/s1. Dataset S1. Fasta files of 75 AQP proteins in soybean. Dataset S2. Fasta files of AQP proteins in *Arabidopsis*, *Phaseolus vulgaris*, *Populus trichocarpa* and *Lotus japonicus*. Dataset S3. Fasta file of the promoter for *GmTIP2;6* in soybean. Table S1. Gene expression numbers for *GmAQPs* and *AtAQPs* in response to heat stress and hormone signals. Table S2. List of primers used in this study. Figure S1. Predicted structures of 75 AQP proteins in soybean. Figure S2. Conserved amino acid residues (NPA motifs, ar/R filter, FPs) and TM domains of PIPs in soybean. Figure S3. Conserved amino acid residues (NPA motifs, ar/R filter, FPs) and TM domains of TIPs in soybean. Figure S4. Conserved amino acid residues (NPA motifs, ar/R filter, FPs) and TM domains of NIPs in soybean. Figure S5. Conserved amino acid residues (NPA motifs, ar/R filter, FPs) and TM domains of SIPs in soybean. Figure S6. Conserved amino acid residues (NPA motifs, ar/R filter, FPs) and TM domains of XIPs in soybean. Figure S7. Distribution of *cis*-acting elements in 75 soybean *AQP* gene promoters. 1500 bp adjacent to the AQP coding sequence. The elements are represented by different colors. The scale bar represents 500 bp. Figure S8. Expression profiles of *Arabidopsis AQP* genes in response to heat stress. Differential sub-family *AQP* gene expression in response to heat stress across eight time points (0.25, 0.5, 1, 3, 4, 6, 12 and 24 hour) in two tissues (roots and shoots). Differences in gene expression changes are shown in color in the green-red scale. Figure S9. Expression profiles of *Arabidopsis AQP* genes in response to ABA, ACC, and MeJA signals. Differential sub-family *AQP* gene expression in response to ABA, ACC, and MeJA hormones across three time points (0.5, 1, and 3 hour). Differences in gene expression changes were shown in color in the green-red scale. Figure S10. Restrictive enzyme sites of pBI121 for promoter vector construction. The *Hind* III site upstream of the *CaMV 35S* promoter was replaced by three continuous sites (*Hind* III, *Pst* I, and *Sal* I).

## Abbreviations

AQP	aquaporin
ABA	abscisic acid
ACC	l-aminocyclopropane-l-carboxylic acid
SA	salicylic acid
MeJA	methyl jasmonate
qRT-PCR	quantitative real-time PCR
GUS	beta-glucuronidase
