# Supplementary figures and images for: Investigation of the AQP Family in Soybean and the Promoter Activity of TIP2;6 in Heat Stress and Hormone Responses
Source: Int J Mol Sci. 2019 Jan 10;20(2):262. doi: 10.3390/ijms20020262 (PMC6359280; doi:10.3390/ijms20020262)

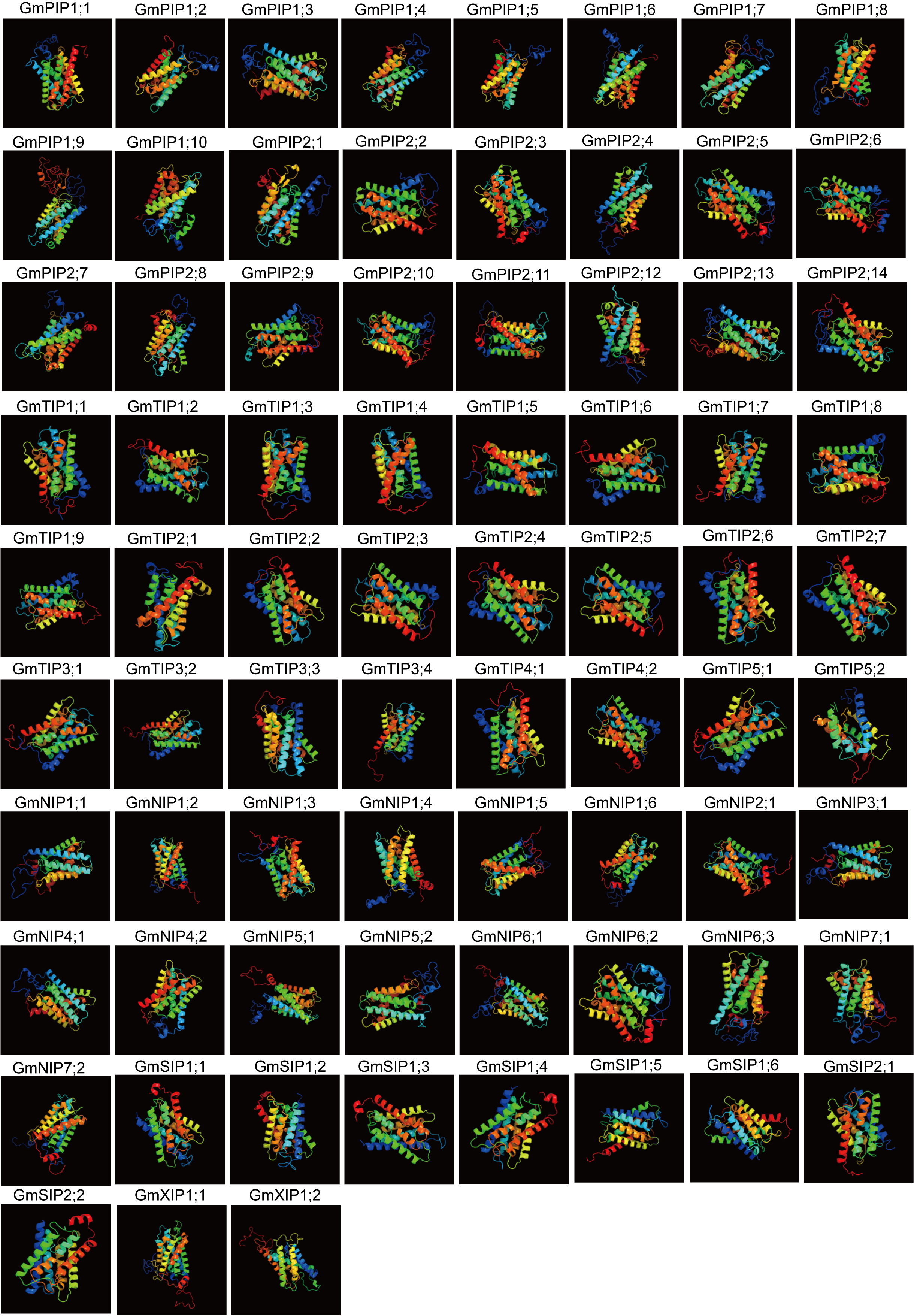

Supplement: Supplementary file 1 [file ijms-20-00262-s001.zip › ijms-415151-Supplementary Materials/Figure S1.tif]

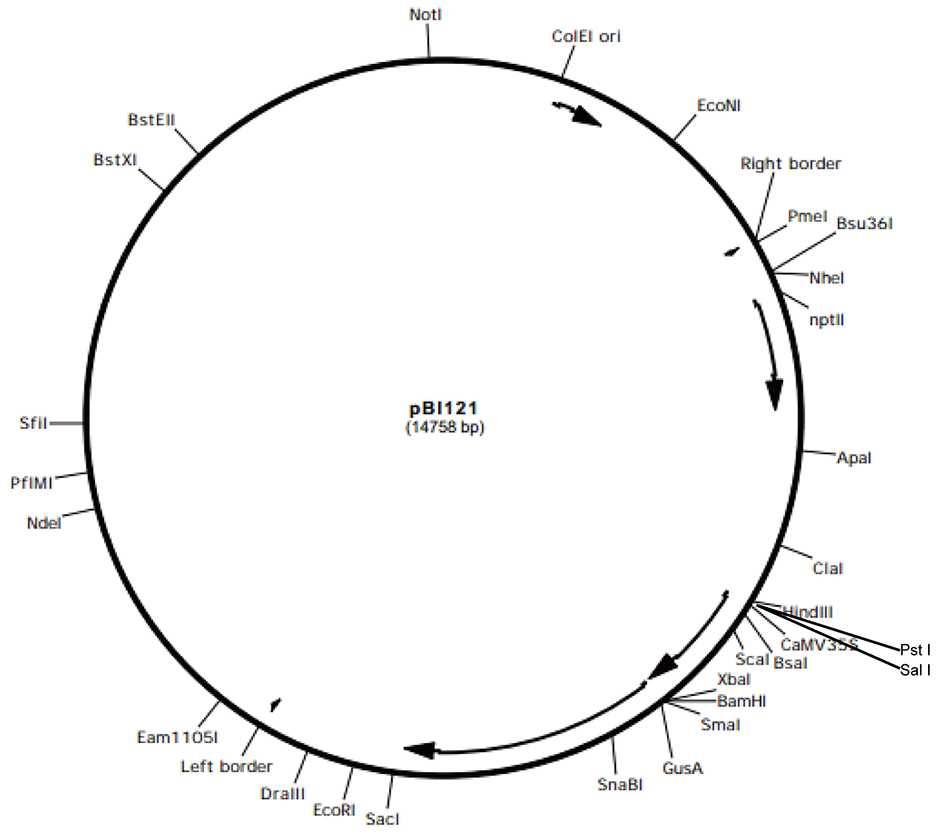

Supplement: Supplementary file 1 [file ijms-20-00262-s001.zip › ijms-415151-Supplementary Materials/Figure S10.tif]

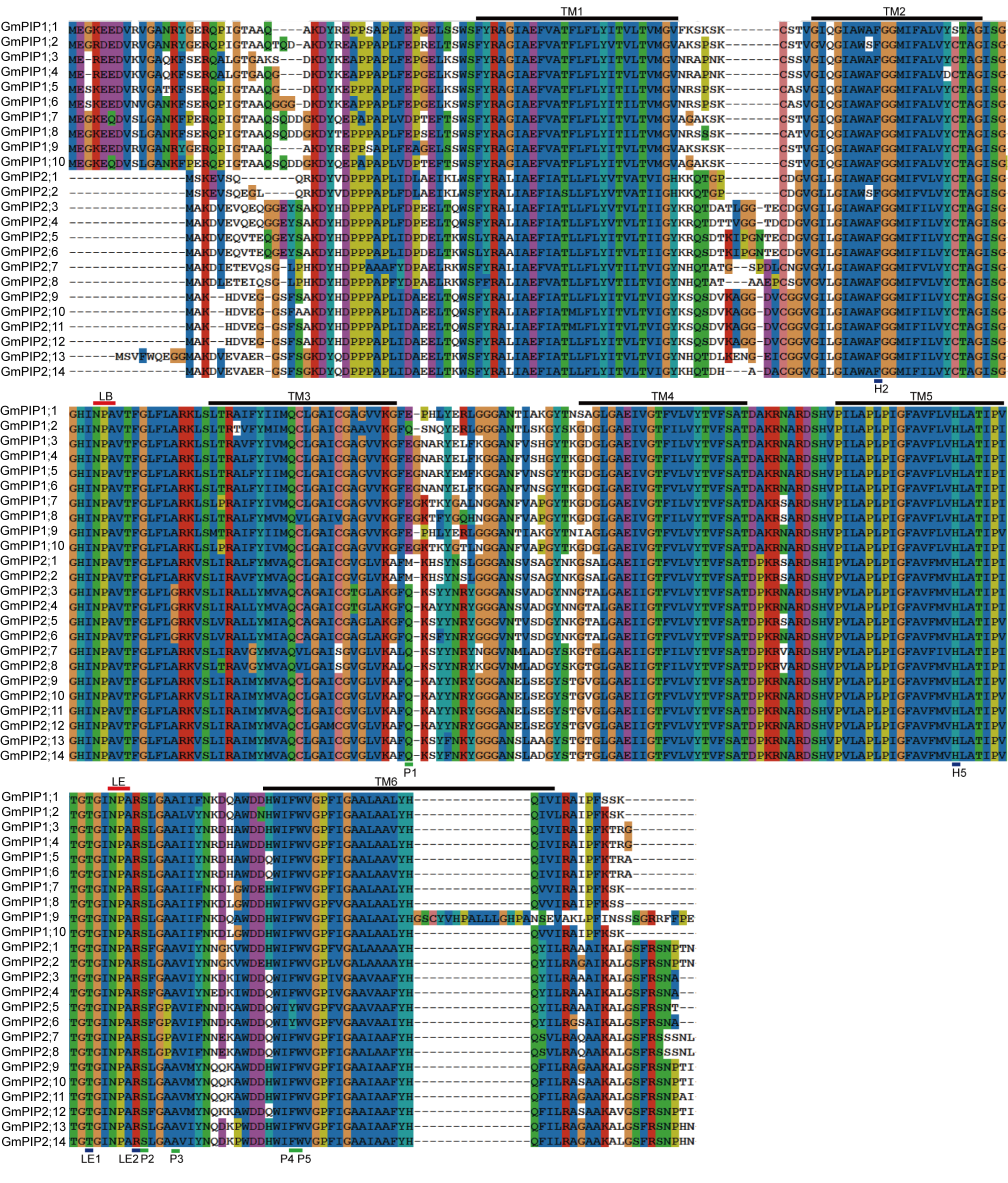

Supplement: Supplementary file 1 [file ijms-20-00262-s001.zip › ijms-415151-Supplementary Materials/Figure S2.tif]

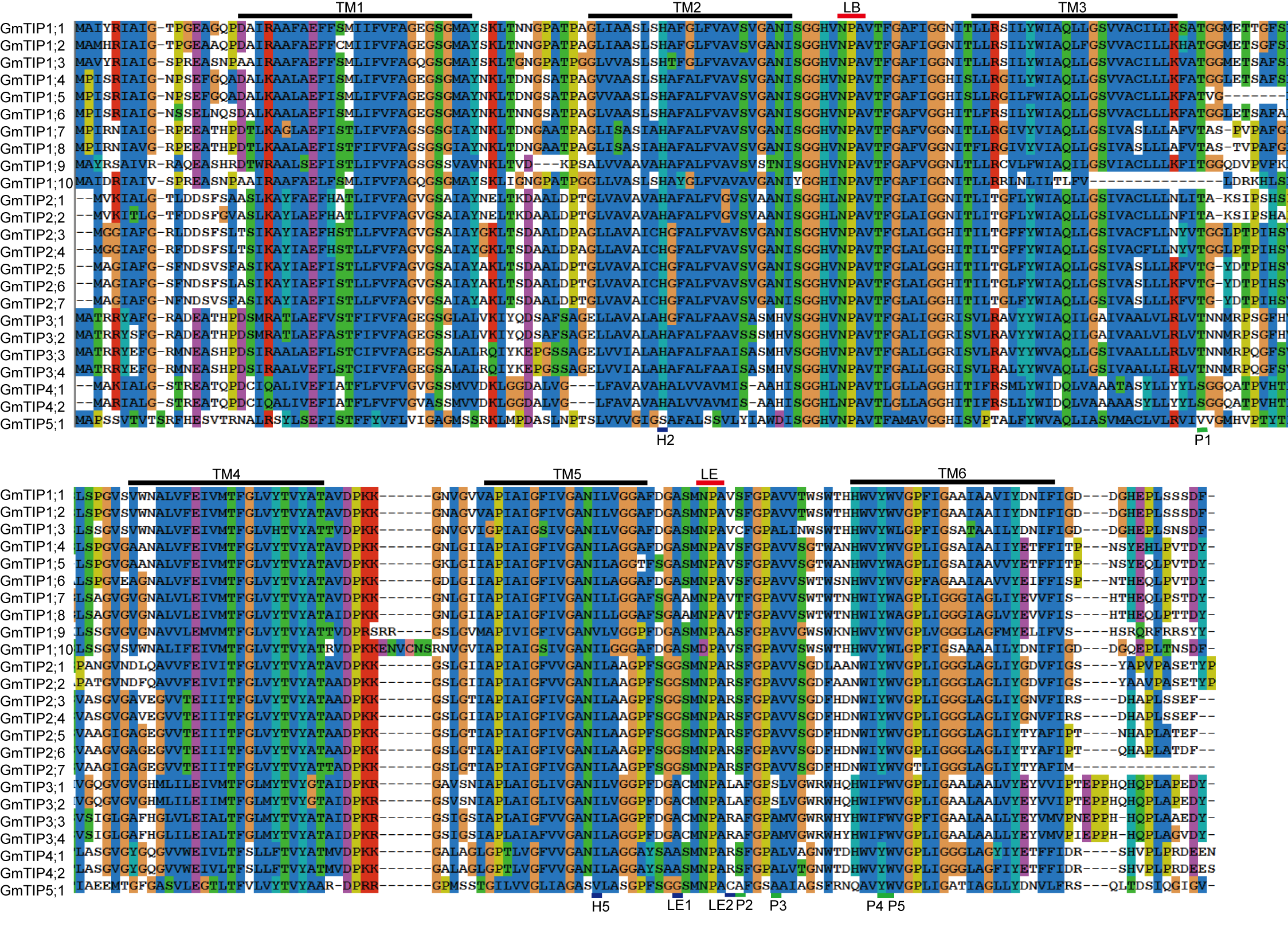

Supplement: Supplementary file 1 [file ijms-20-00262-s001.zip › ijms-415151-Supplementary Materials/Figure S3.tif]

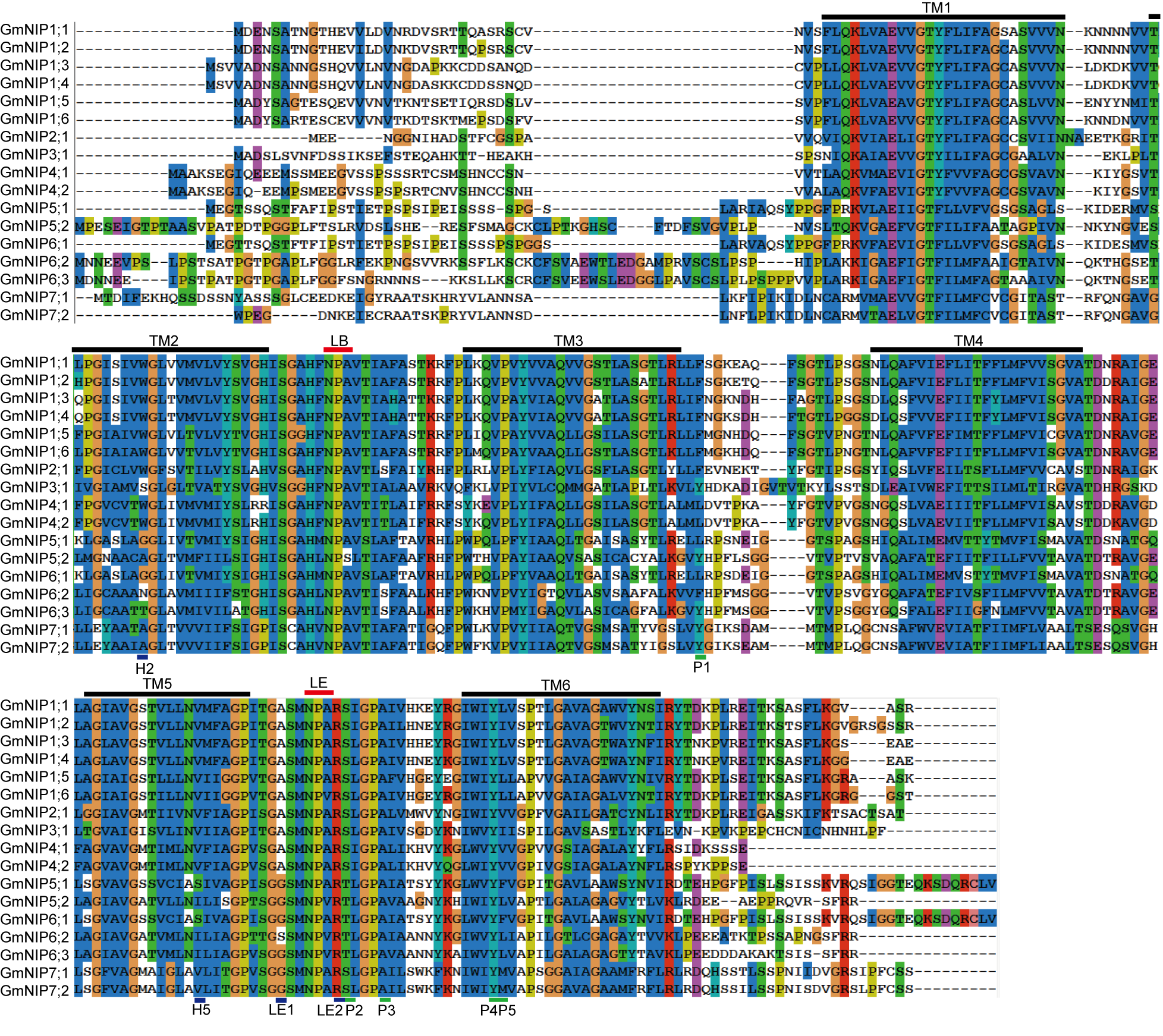

Supplement: Supplementary file 1 [file ijms-20-00262-s001.zip › ijms-415151-Supplementary Materials/Figure S4.tif]

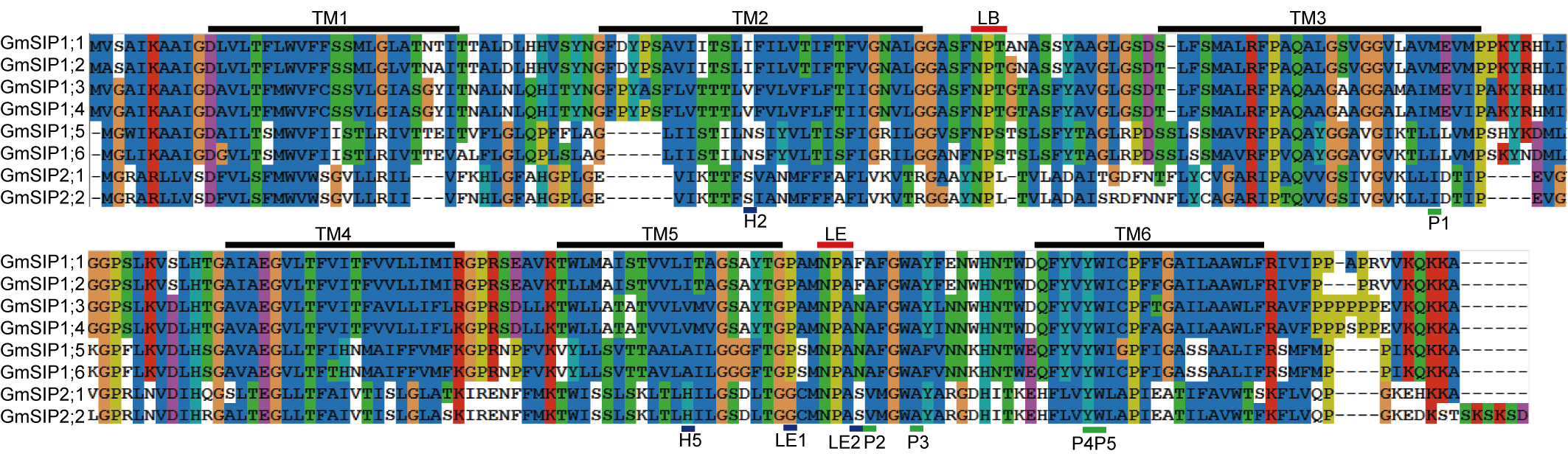

Supplement: Supplementary file 1 [file ijms-20-00262-s001.zip › ijms-415151-Supplementary Materials/Figure S5.tif]

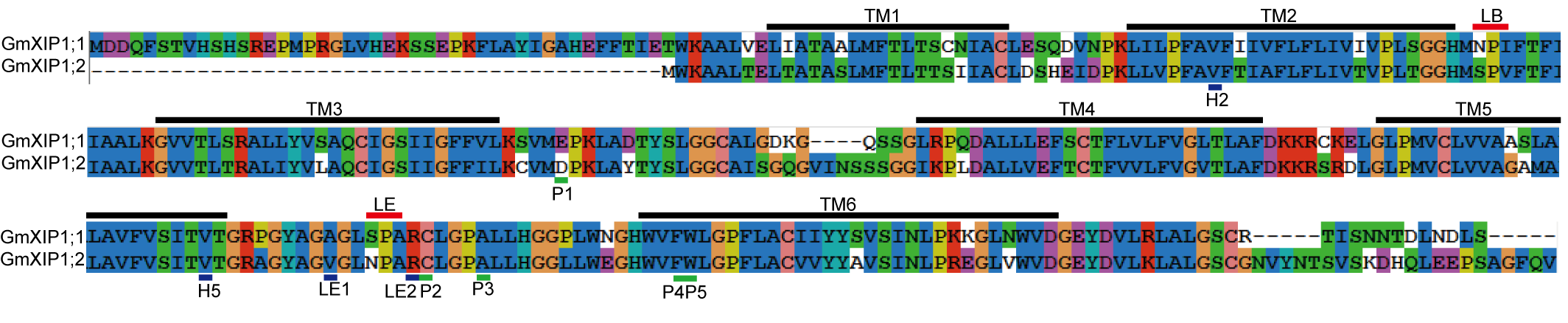

Supplement: Supplementary file 1 [file ijms-20-00262-s001.zip › ijms-415151-Supplementary Materials/Figure S6.tif]

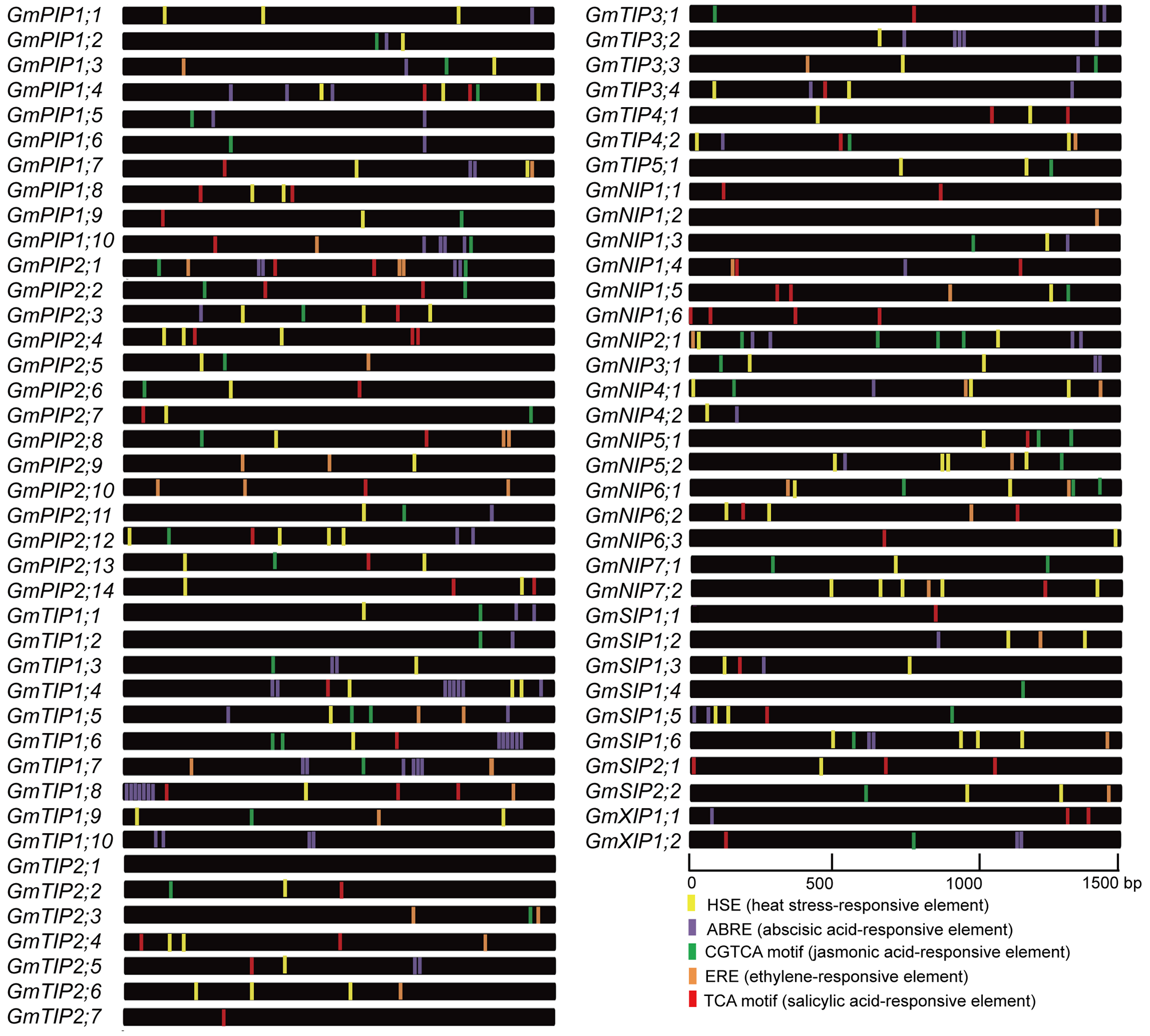

Supplement: Supplementary file 1 [file ijms-20-00262-s001.zip › ijms-415151-Supplementary Materials/Figure S7.tif]

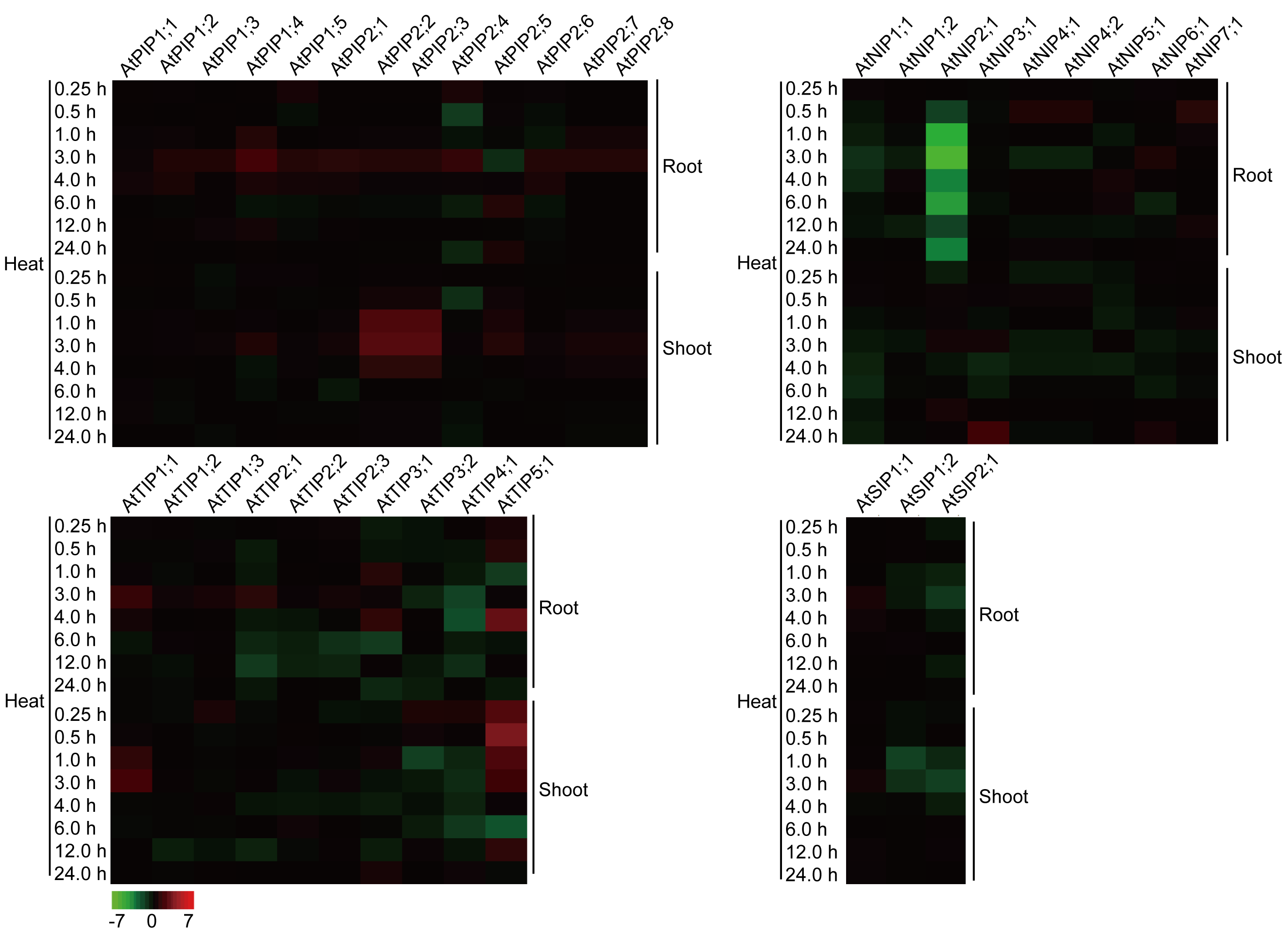

Supplement: Supplementary file 1 [file ijms-20-00262-s001.zip › ijms-415151-Supplementary Materials/Figure S8.tif]

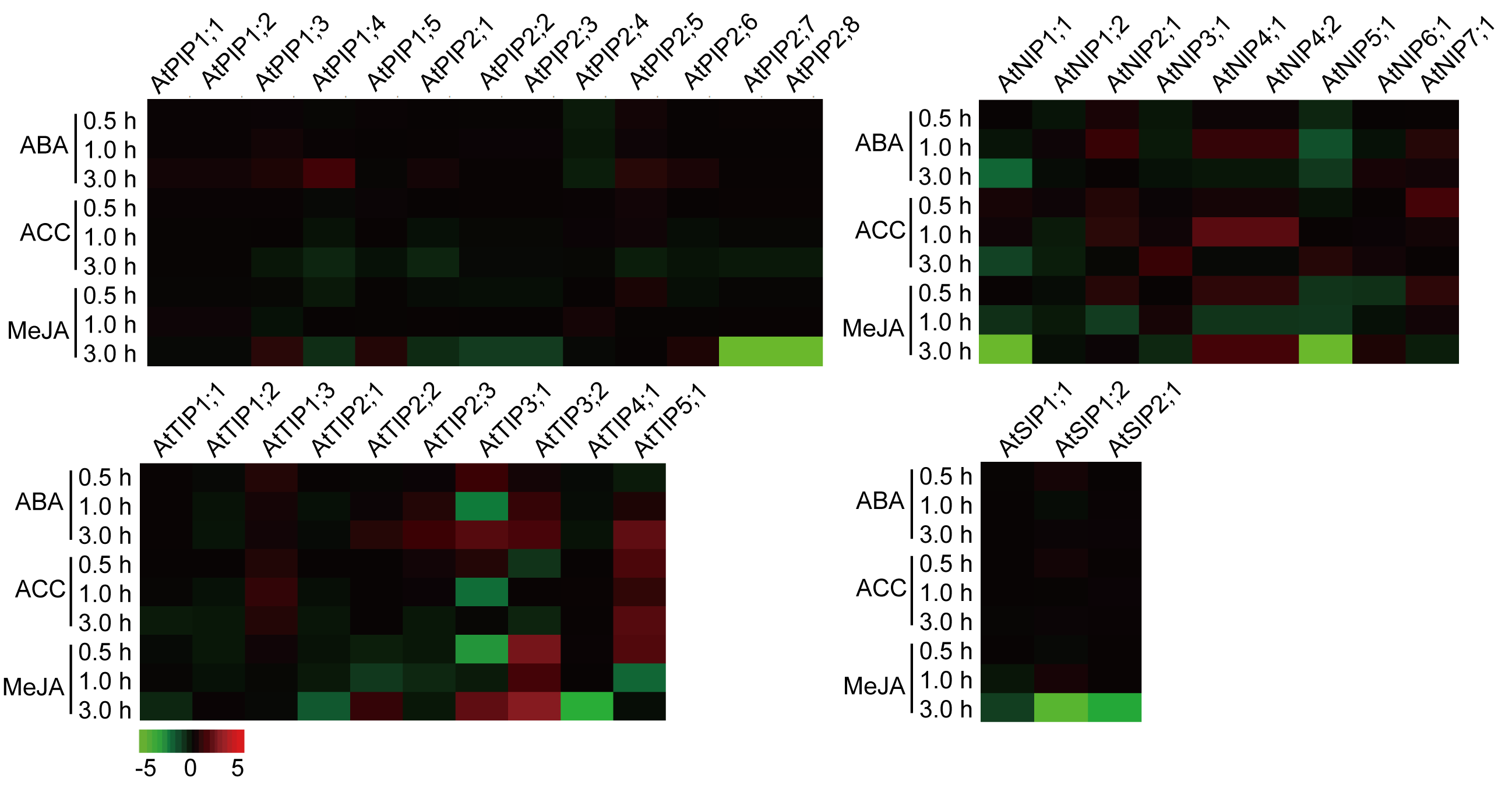

Supplement: Supplementary file 1 [file ijms-20-00262-s001.zip › ijms-415151-Supplementary Materials/Figure S9.tif]
